# Multi-level block permutation

**DOI:** 10.1016/j.neuroimage.2015.05.092

**Published:** 2015-12

**Authors:** Anderson M. Winkler, Matthew A. Webster, Diego Vidaurre, Thomas E. Nichols, Stephen M. Smith

**Affiliations:** a Oxford Centre for Functional MRI of the Brain, University of Oxford, Oxford, UK; b Oxford Centre for Human Brain Activity, University of Oxford, Oxford, UK; c Department of Statistics & Warwick Manufacturing Group, University of Warwick, Coventry, UK

**Keywords:** Permutation inference, Multiple regression, General linear model, Repeated measurements

## Abstract

Under weak and reasonable assumptions, mainly that data are exchangeable under the null hypothesis, permutation tests can provide exact control of false positives and allow the use of various non-standard statistics. There are, however, various common examples in which global exchangeability can be violated, including paired tests, tests that involve repeated measurements, tests in which subjects are relatives (members of pedigrees) — any dataset with known dependence among observations. In these cases, some permutations, if performed, would create data that would not possess the original dependence structure, and thus, should not be used to construct the reference (null) distribution. To allow permutation inference in such cases, we test the null hypothesis using only a subset of all otherwise possible permutations, i.e., using only the rearrangements of the data that respect exchangeability, thus retaining the original joint distribution unaltered. In a previous study, we defined exchangeability for blocks of data, as opposed to each datum individually, then allowing permutations to happen within block, or the blocks as a whole to be permuted. Here we extend that notion to allow blocks to be nested, in a hierarchical, multi-level definition. We do not explicitly model the degree of dependence between observations, only the lack of independence; the dependence is implicitly accounted for by the hierarchy and by the permutation scheme. The strategy is compatible with heteroscedasticity and variance groups, and can be used with permutations, sign flippings, or both combined. We evaluate the method for various dependence structures, apply it to real data from the Human Connectome Project (HCP) as an example application, show that false positives can be avoided in such cases, and provide a software implementation of the proposed approach.

## Highlights

• The presence of structured, non-independent data affects simple permutation testing.
• Modelling full dependence obviated through definition of variance groups (minimal assumptions).
• Implementation based on shuffling branches of a tree-like (hierarchical) structure.
• Validity demonstrated with simulations, and exemplified with data from the HCP.

## Introduction

In the context of hypothesis testing using the general linear model (glm) (Scheffé, 1959, Searle, 1971), permutation tests can provide exact or approximately exact control of false positives, and allow the use of various non-standard statistics, all under weak and reasonable assumptions, mainly that the data are *exchangeable* under the null hypothesis, that is, that the joint distribution of the error terms remains unaltered after permutation. Permutation tests that compare, for instance, groups of subjects, are of great value for neuroimaging (Holmes et al., 1996, Nichols and Holmes, 2002), and in Winkler et al. (2014), extensions were presented to more broadly allow tests in the form of a glm, and also to account for certain types of well structured non-independence between observations, which ordinarily would preclude the use of permutation methods. This was accomplished by redefining the basic exchangeable unit from each individual datum to blocks of data, i.e., rather than asserting exchangeability across all observations of a given experiment, blocks of exchangeable units are defined; these *exchangeability blocks* (ebs) can be rearranged as a whole (*whole-block exchangeability*), or the observations within block can be shuffled among themselves (*within-block exchangeability*), using either permutations, sign flippings, or permutations combined with sign flippings.

In the same work, the *G*-statistic, a generalisation over various commonly used statistics, including the *F*-statistic, was proposed. *G* is robust to known heteroscedasticity (i.e., the situation in which the variances are known to be not equal across all observations, which can be then classified into variance groups) and can be used with the glm, ensuring that pivotality^1^ is preserved, a crucial requisite for exact control over familywise error rate (fwer) using the distribution of the most extreme statistic (Westfall and Young, 1993), as needed in many neuroimaging studies. Indeed, the use of ebs allows for variances to be heterogeneous, provided that the groups of observations sharing the same variance (i.e., *variance groups*, vgs) (Woolrich et al., 2004) are compatible with the ebs; specifically, for within-block exchangeability the vgs must coincide with the blocks, and for whole-block exchangeability they must include one or more observations from each block in a consistent order.

This arrangement, using a statistic that is robust to heteroscedasticity, the use of variance groups, and the imposition of restrictions on exchangeability through the use of ebs, allows inference on various designs that, otherwise, would be much more difficult to do non-parametrically. These designs include paired tests, longitudinal designs, and other common tests that involve repeated measurements. However, certain study designs, despite exhibiting well-structured dependence between observations, still cannot be accommodated in the above framework. This occurs when the overall covariance structure is known, but its exact magnitude is not. An example occurs when multiple measurements per subject are performed in more than one session, with more than one measurement per session: the measurements within session may be exchangeable, but not across sessions. Another example is for studies using siblings, such as designs using discordant sib-pairs (in which only one sibling is affected by a given disorder), or using twins: permutations that disrupt the constitution of any sibship cannot be performed, as this would violate exchangeability.

Studies such as these are relatively common, notably those that involve siblings. However, whereas in classical twin designs the central objective is to quantify the fraction of the variation in a measurement (trait) that can be explained by the familial relationship between subjects after potential confounds have been taken into account, a quantity known as *heritability*, here the concern is with a general linear model, and the objective is to test the influence of explanatory variables on the observed data. In other words, the interest lies on the relationship between the covariates and the main trait, while the non-independence between observations, which is a feature of interest in a heritability study, is here a form of nuisance that imposes restrictions on exchangeability for permutation inference for the glm.

Rather than inadvertently breaking these restrictions, here we propose to test the null hypothesis using a subset of all otherwise possible permutations, only allowing the rearrangements that respect exchangeability, thus retaining original joint distribution unaltered.^2^ As in our previous work, we treat observations or entire blocks of data as weakly exchangeable, but here we further extend the definition of ebs to allow more complex designs to be addressed. This is accomplished through the use of *multi-level exchangeability blocks*, in which levels consist of *nested* blocks; for each such block the state of within- or whole-block exchangeability can be specified. The blocks are defined hierarchically, based on information about the dependence within data, but not requiring the modelling of the actual dependency. Even though the possibility of using nested blocks was anticipated in Winkler et al. (2014) (“Whole-block and within-block can be mixed with each other in various levels of increasing complexity”, page 386), nothing further was studied or presented at the time. Here we provide a comprehensive description of the approach, investigate its performance, its power, and present an applied example using the data structure of the ongoing Human Connectome Project (hcp). In the Appendix A, we present an implementation strategy.

## Theory

### Terminology

When contrasting the method described in this article with simple data rearrangement, various terms could be adopted: *single-level* vs. *multi-level* block shuffling, emphasising the levels of relationship between observations; *unrestricted* vs. *restricted*, emphasising the imposition of restrictions on how the data are allowed to be rearranged at each shuffling; *free* vs. *tree* shuffling, emphasising the tree-like structure of the relationships between observations that allow shuffling. All these terms have equivalent meaning in the context of this article, and are used interchangeably throughout. The generic terms *shuffling* and *rearrangement* are used when the distinction between permutations, sign flippings or permutations with sign flippings is not relevant.

### Notation

We consider a glm that can be expressed as **Y** = **M*ψ*** + **ϵ**, where **Y** is the *N* × 1 vector of observed data, **M** is the full-rank *N* × *r* design matrix that includes explanatory variables (i.e., effects of interest and possibly nuisance effects), ***ψ*** is the *r* × 1 vector of *r* regression coefficients, and **ϵ** is the *N* × 1 vector of random errors. Estimates for the ***ψ*** can be computed by ordinary least squares, i.e., $\hat{\psi }=M^{+}Y$, where the superscript (^+^) denotes a pseudo-inverse. One generally wants to test the null hypothesis that a given combination (contrast) of the elements in ***ψ*** equals to zero, that is, H_0_ : **C**′***ψ*** = **0**, where **C** is a *r* × *s* full-rank matrix of *s* contrasts, 1 ≤ *s* ≤ *r*. The commonly used *F* statistic can be computed as usual and used to test the null hypothesis. When *s* = 1, the Student's *t* statistic can be computed as $t=sign\left(\hat{\psi }\right)\sqrt{F}$. A p-value for the statistic is calculated by means of shuffling the data, the model, the residuals, or variants of these (Winkler et al., 2014, Table 2). In any of these cases, to allow rearrangements of the data, some assumptions need to be made: either of *exchangeable errors* (EE) or of *independent and symmetric errors* (ISE). The first allows permutations, the second sign flippings; if both are available for a given model, permutations and sign flippings can be performed together. These rearrangements are represented by permutation and/or sign flipping matrices **P**, and the set of all such matrices allowed for a given design is denoted as P.

**Fig. 1:**
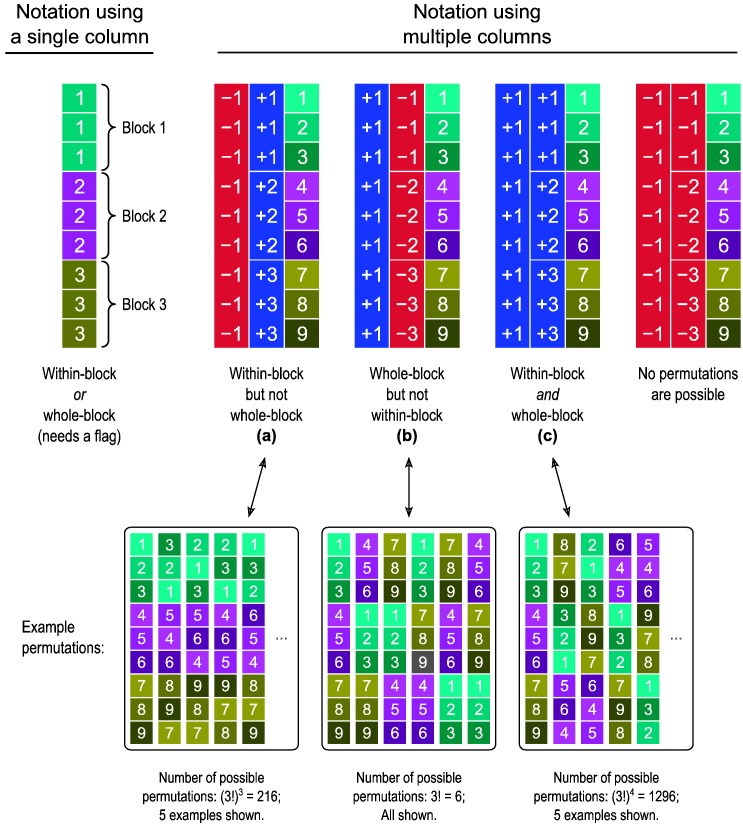
Different notations for the specification of exchangeability blocks; in this example, 3 blocks of 3 observations each. Left: In a single-column notation, each block has its index (here 1, 2, and 3, shown in different, random colours for clarity), and either within- or whole-block exchangeability are possible, but not both simultaneously. The specification of which kind of shuffling is to be done requires extra information, as a flag passed to the algorithm that permutes the data. Right: In a multiple-column notation, that information is encoded by virtue of the indices having a sign indicating whether the exchangeable units of a block at a given level should be shuffled as a whole (+) or kept fixed (−); these are shown respectively in blue and red. The signs define whether it is possible to perform rearrangements within-block, or of the blocks as a whole, or both. The rightmost example serves only to illustrate the notation, and is not useful in practice as all the observations would need to remain still. The letters (a) through (c) refer to the visual representations in Fig. 2. Bottom: Example permutations are shown, with the observation indices coloured for clarity.

At its simplest, the ebs for within- or whole-block exchangeability can be identified or represented by a set of indices {1,2…,*B*}, one for each of the *B* blocks. A vector of size *N* × 1, can be used to indicate to which eb each observation from **Y** belongs (Fig. 1, *left*); an extra flag is passed to the shuffling algorithm (such as the randomise algorithm) to indicate whether the rearrangements of the data should happen as within- or as whole-block. While this notation probably covers the majority of the most common study designs, it allows only within- *or* whole-block, but not *both* simultaneously; in other words, if in a study the observations can be permuted within block, and the blocks as a whole can also be permuted, such notation does not convey all possibilities for reorganising the data while preserving their joint distribution unaltered, and algorithms would perform fewer shufflings than those that are effectively allowed.

This can be addressed by extending the notation from a single column to a multi-column array, allowing nested ebs to be defined, such that blocks can contain sub-blocks, in a hierarchical fashion, and where each column represents a level; we use the leftward columns to indicate higher, and rightward to indicate lower levels. More columns alone, however, are not sufficient, because at each level, shufflings of observations or of sub-blocks can be allowed within-block, or the blocks at that level can be shuffled as a whole. Hence to discriminate between one type or the other, we use negative indices to indicate that the exchangeable units at the level immediately below should not be permuted, and positive indices indicate that shuffling of these units is allowed (Fig. 1, *right*). The exchangeable units can be sub-blocks, which can contain yet other sub-blocks, or observations if the next level immediately below is the last.

These two notations, i.e., using single- or multi-column indices, do not represent mathematical entities, and are not meant to be used for algebraic manipulation; rather, these notations are shorthand methods to represent structured relationships between observations. The covariance structure prevents unrestricted shuffling from being considered, but it often permits shufflings to happen in a certain orderly manner that preserves the joint distribution of the data. These notations are to be used by the algorithm that performs the test to construct the permutation and/or sign flipping matrices, which then can be used to effectively disarrange the model to construct the distribution of the statistic under the null hypothesis.

### Visual representation

**Fig. 2:**
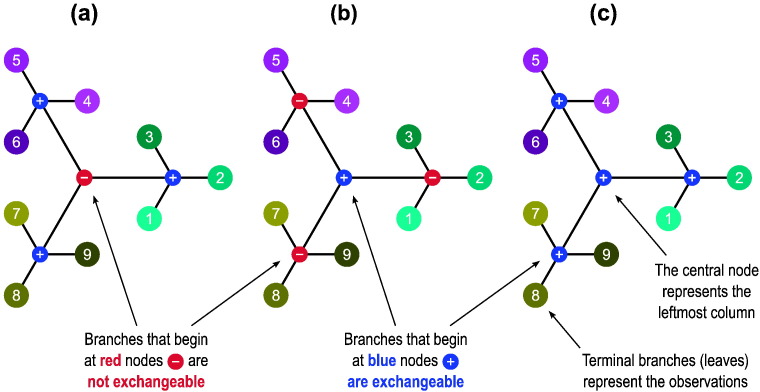
Visual representations for the multi-level notation in the examples (a)–(c) from Fig. 1, and using the same colour scheme. The levels can be depicted as branching from a central (top) node, akin to a tree in which the most peripheral elements (leaves) represent the observations. The nodes from which the branches depart can be labelled as allowing permutations (+) or not (−), shown respectively here in blue and red colours.

The notation using multiple columns encapsulates all the information necessary not only for the rearrangements to be constructed, but also to depict the relationships between the observations in a tree-like diagram, highlighting their hierarchy, as shown in Fig. 2. Branches can only be shuffled with each other if their size and internal covariance structure are perfectly identical; this information is contained in the signs and indices used to represent each block: positive indices (shown in blue) allow them to be permuted; negative (in red) prevents permutation and keeps the branches in their fixed positions. The permutation of branches at lower levels (when these exist) is controlled by the nodes at these lower levels, independently from those at higher levels or within the same level.

**Fig. 3:**
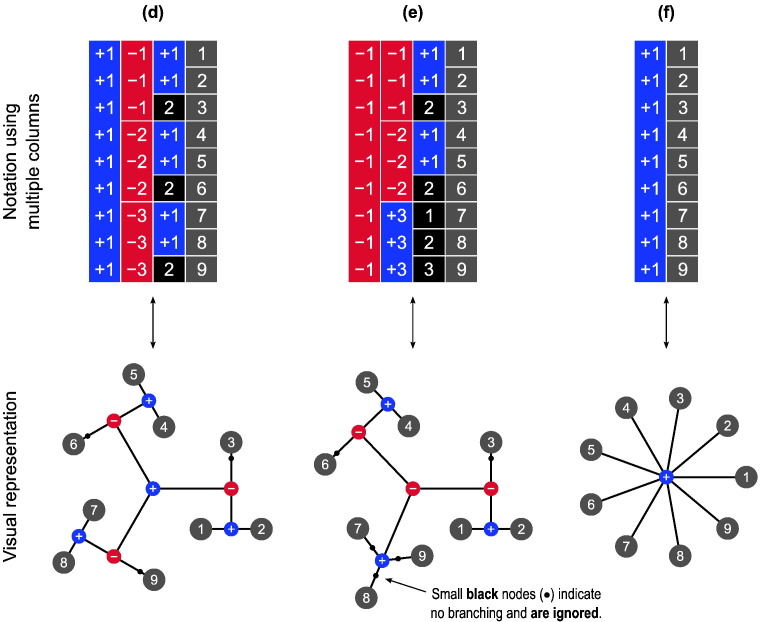
The multi-level definition of blocks allows more complex relationships between observations. *Left*: Three blocks of identical structure (2nd column) can be shuffled as a whole (as indicated by the positive indices in the 1st column); within each (3rd column), only two out of their three constituting observations can be swapped (1 and 2, 4 and 5, and 7 and 8), whereas the third on each (3, 6 and 9) cannot; levels for these last branches are completed with blocks for which the sign has no meaning (in black), as they remain unaltered towards the next level (4th column), and represent no actual branching. In the visual representation, these black blocks are shown as small black dots on continuous branches. This example could represent 3 sets of siblings, each composed of a pair of monozygotic twins and a third non-twin. *Centre*: An example showing that it is possible to mix types of blocks in the same level (2nd column). As shown, the first two blocks in the 2nd column cannot be swapped despite similar coding, and neither of these can be permuted with the third, which has a different structure consisting of three observations (7, 8 and 9) that can be shuffled freely. This example could represent 3 sets of siblings, the first a pair of monozygotic twins and a non-twin, the second a pair of dizygotic twins and a non-twin (if certain environmental effects are considered), and the third a set of three non-twin siblings. *Right*: The same notation can also accommodate simple designs. Here all 9 observations can be permuted without restrictions on exchangeability.

Using the tree diagram, it becomes clear that the terms “within-block” and “whole-block”, that have been used so far to describe exchangeability and permutation strategies, become no longer necessary, as either the branches can be shuffled, or they cannot. It is also helpful in emphasising that more complicated designs can be considered using multi-level blocks, in which even the distinction between within- and whole-block is softened, as each level in the multi-column notation is not restricted to contain purely positive or negative indices restricting (or not) the shuffling of their constituent sub-blocks (branches). These can be present alongside each other if immediately below a level in which shuffling is not allowed, such that some branches may be allowed to be shuffled, whereas others are not. It may also be the case that some levels need to be included in the notation only so that the number of levels remains the same across all branches of the tree, from the top node to the most distal (leaves), without affecting the construction of P, but ensuring that the notation can be stored, without gaps, in a two-dimensional array; in the visual representation these are shown as small, sign-less, black nodes. Fig. 3 (*left* and *centre*) exemplifies these cases. Although the multi-column notation and the corresponding tree can become very complex, the simple, unrestricted exchangeability can also be accommodated, as shown in Fig. 3 (*right*).

### Variance groups and the *G*-statistic

When the variances can be assumed to be the same throughout the sample, the classical *F* and the Student's *t* statistics can be used; these statistics have sampling distributions that do not depend on any unknown population parameters, but solely on the degrees of freedom, i.e., these are pivotal statistics. However, if homoscedasticity cannot be assumed, although *F* and *t* can still be used with permutation tests in general, they cannot be used to correct for multiple testing using the distribution of the most extreme statistic. The reason is that under heteroscedasticity, these statistics cease to be pivotal, and follow instead distributions that depend on the heterogeneous variances for the different groups of observations, causing them to be no longer adequate for fwer correction. Instead, a statistic that is robust to heteroscedasticity is necessary.

The *G*-statistic (Winkler et al., 2014) was proposed to address this concern; this statistic is a generalisation of various other well established statistics, including *F* and *t*, as well as the *v*-statistic used for the classical Behrens–Fisher problem. The definition of the variance groups used to calculate *G* is based on knowledge about the data, and such groups need to be constructed together with the definition of the blocks. However, vgs and ebs represent different concepts; although they may coincide for simple designs, they do not need to. The ebs are used to indicate sets of observations that must remain together in every permutation due to having a non-diagonal *covariance* structure, and are used by the permutation algorithm to rearrange the data many times to build the empirical distribution. The vgs, however, are used to indicate sets of observations that possess the same *variance*, and are used to estimate the sample variance(s) when computing the statistic. Despite the distinction, any pair of observations that have the possibility of being swapped according to the eb structure must be in the same vg; observations in different variance groups cannot be permuted as that would modify the joint distribution, thus violating exchangeability.

**Fig. 4:**
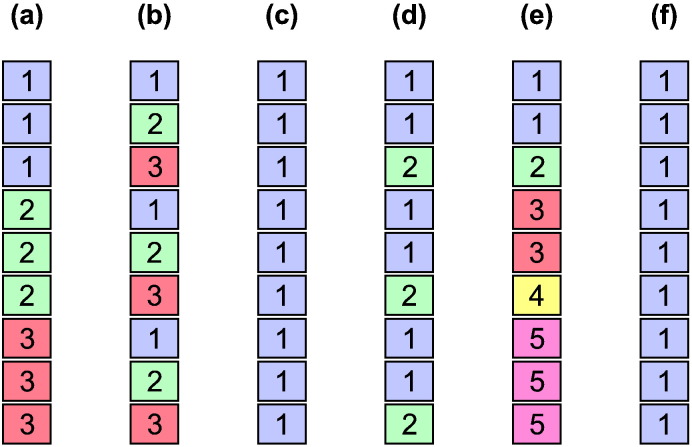
Variance groups defined from the exchangeability blocks (a)–(c) shown in Fig. 1, and (d)–(f) in Fig. 3. These are the most restrictive configurations for the vgs that are possible given the structure imposed by the ebs. If, however, despite the covariance structure between observations, their variances are known to be or can be assumed to be homogeneous, some or all of these groups can be merged, with the additional benefit of improving the variance estimates. Alternatively, the groups can be entirely replaced by a different definition if additional information from the variance of the data is available. In (e), note two groups with only one observation each; see the main text for details.

For simple within-block permutation, the most restrictive configuration for the variance groups, that is, the configuration in which fewer observations need to be assumed to share the same variance, is such that each block corresponds to its own vg. For simple whole-block permutation, on the other hand, the first observation from each block, together, constitute a vg, the second observation from each block, together, another vg, and so forth. The minimum set of variance groups for more complicated designs can be derived from the configuration of the exchangeability blocks; examples are shown in Fig. 4. The stringency of this definition lies in that, depending on the configuration of the ebs, each vg can contain only the smallest possible number of observations that can be assumed to have the same variance given the covariance structure imposed by the blocks. Such definition can, however, be relaxed by merging these minimum groups whenever homoscedasticity across more than one vg can be considered, while retaining the ebs unaltered. Whether merger, or any other definition, for the vgs should be sought for a given design may depend on information about the data or on the design itself. For a simple paired *t*-test, for instance, although each pair could in principle constitute a vg on its own, homogeneous variances can fairly well be assumed, with the benefit of much better variance estimates than would be obtained with groups of two sole observations.

Regardless of which strategy is used to define the variance groups, and irrespective of the indices used to represent each of them, the column vector containing these indices must be invariant with respect to the permutations that are allowed for a given design. In other words, let **v** be the column vector of length *N* containing the indices that represent each variance group, such as those in Fig. 4. For any permutation matrix P∈P, **Pv** = **v**, that is, **v** is a common eigenvector for all permutation matrices in P. Any permutation that breaks this equality must not be used to test the null hypothesis, as this would mix observations that belong to different vgs, thus violating exchangeability. Likewise, a definition of groups that does not meet this criterion must not be used.

### Number of permutations

With the multi-level block permutation strategy, the rules to calculate the number of permutations are similar, yet more general than in the case of a single level that could be represented with a single-column notation (Winkler et al. 2014). The number still depends on the number of repeated rows in the design matrix for methods as Manly and ter Braak (Manly, 1986, ter Braak, 1992) or, for methods as Draper–Stoneman and Freedman–Lane (Draper and Stoneman, 1966, Freedman and Lane, 1983), on the number of repeated rows across only the columns that are tested in the contrast after the model has been partitioned into effects of interest and nuisance effects.

Once the tree has been constructed, for the ee assumption, and in the absence of repetitions in the design as described above, the number of permutations can be calculated separately for each node in which shuffling is allowed as *B* !, with *B* denoting the number of branches that begin at that node. If however, there are branches with identical structure and containing the repetitions in the design matrix, the number of possible permutations for that node is then *B* !/∏_*m* = 1_^*M*^*B*_*m*_ !, where *M* is the number of unique branches beginning at that node, and *B_m_* the number of times each of the *M* unique branches begins at that node. The number of permutations for nodes that cannot be permuted is simply 1, that is, no permutation. With the number of permutations at each node calculated, the overall number of possible permutations for the whole design (whole tree) is the product of the number of possible permutations for all the nodes.

For ise, the number of sign flippings at the highest node in which shuffling is allowed is 2^*B*^, and 1 for all other nodes that lie below (distal) in the hierarchy. For the nodes in which shuffling is not allowed, the number of possible flips is 1, that is, no sign flippings are allowed, but it can still be higher than 1 for the nodes that lie below in the hierarchy. Unlike with permutations, the eventual presence of repeated elements in the design matrix does not affect the number of possible sign flippings. The number of possible sign flippings for the whole design is the product of the number of sign flippings for all the nodes.

When both ee and ise assumptions are valid for a given design, permutations can happen with sign flipping, and the total number of possible rearrangements is just the product of the number of permutations with the number of sign flippings. Regardless of the kind of shuffling strategy adopted, the number of possible rearrangements can be extremely large, even for sample sizes of relatively moderate size. Not all of them need to be performed for the test to be valid and sufficiently exact; a random subset of all possible rearrangements can be sufficient for accurate hypotheses testing.

### Power and outliers

The set of all rearrangements that can be performed while respecting the structure of the data is termed the *permutation space* (Pesarin and Salmaso, 2010). The restrictions imposed by the ebs cause this space to be reduced, sometimes considerably, as none of the rearrangements that would violate exchangeability are performed. If the restrictions are such that the permutation space is not a representative, uniform sample from what the space would be without such restrictions, power may be reduced. In the Evaluation method section we assess various configurations for the multi-level ebs and their impact on the ability to detect true effects.

For the same reason, even though most permutation strategies tend to be robust to outliers (Anderson and Legendre, 1999), the dependence structure and the multi-level blocks may amplify the effect of their presence, with results that may be difficult to predict, either in terms of conservativeness or anticonservativeness. Such problems may be minimised by providing some treatment of these extreme values; some possible remedies include censoring, trimming, replacement for ranks or quantiles, conversion of quantiles to a normal distribution, and robust regression.

## Evaluation method

### Error rates and power

**Fig. 5:**
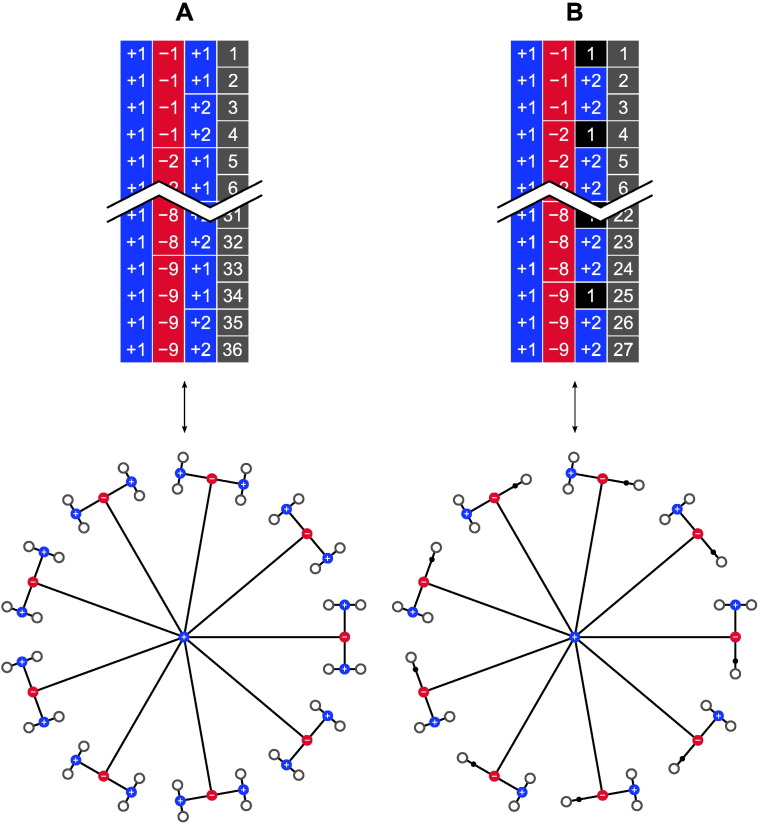
The two dependence structures, a and b, used to assess error rates and power. *Top*: Multi-level block definition. *Bottom*: Visualisation as a tree diagram.

Two dependence structures, named datasets a and b, were simulated to evaluate the permutation strategy. Both use mixtures of levels that can or that cannot be shuffled. For the dataset a, *N* = 36 observations were simulated, grouped into nine exchangeability blocks of four observations each, and each of these further divided into two blocks of two. Not all levels were allowed to be shuffled freely, and the structure is shown in Fig. 5 (*left*). For dataset b, *N* = 27 observations were divided into nine ebs of three observations each; and each of these further divided into two blocks, one with two, and one with one observation, as shown in Fig. 5 (*right*). Although these may appear somewhat artificial for practical use, we wanted examples that would restrict the number of possible shufflings, to test the multi-level strategy in relatively difficult scenarios. The structure in dataset a precisely emulates a twin study with nine sets of siblings, each comprised of a pair of monozygotic twins and a pair of non-twins (or of dizygotic twins). Dataset b uses a similar scheme, but further restricts the possibilities for shuffling by having just one non-twin in each set of siblings.

Using the same notation as in the Notation section, 500 response variables (data vectors **Y**) were simulated for each dataset, using the model **Y** = **M*ψ*** + **ϵ**; each variable might represent, for instance, a voxel or vertex in a brain image. The residuals, **ϵ**, were simulated following either a Gaussian distribution (with zero mean and unit variance), a Weibull distribution (skewed, with scale parameter 1 and shape parameter 1/3, shifted and scaled so as to have expected zero mean and unit variance), or a Laplace distribution (kurtotic, with zero mean and unit variance).^3^ In order to introduce dependence between the residuals, for simplicity and without loss of generality to any study in which there is dependence among the data, including repeated measurements, each observation was treated as if from a participant in a twin study design, as described above, and an *N* × *N* correlation matrix **Ω**, was created using the coefficient of kinship, 2*ϕ*_*ij*_, between subjects *i* and *j* (Jacquard, 1970), such that **Ω** = 2**Φ***h*_**ϵ**_^2^ + **I**(1 − *h*_**ϵ**_^2^), where **Φ** is the matrix with the coefficients *ϕ*_*ij*_, and **I** is the identity matrix. The benefit of constructing the simulations in this way is that the strength of the dependence structure can vary linearly in the interval 0 to 1 using a single parameter, here denoted as *h*_**ϵ**_^2^, which coincides, in quantitative genetics and under certain assumptions, with the heritability of the measurement after explanatory or nuisance variables have been considered. The coefficient of kinship (2*ϕ*_*ij*_) is set to 1 for monozygotic twins, 0.5 full siblings that are not monozygotic twins, 0.25 for half siblings, and 0 for unrelated subjects. For these simulations, we used different values for the heritability of the residuals as *h*_**ϵ**_^2^ = {0, 0.4, 0.8}. To introduce the desired correlation structure, **Ω** was subjected to a Cholesky decomposition such that **Ω** = **L**′**L**, then redefining the residuals as **L**′**ϵ**.

The dependent data, **Y**, were generated by adding the simulated effects, **M*ψ***, to the residuals, **ϵ**, with *ψ* = [*ψ*_1_ 0]′, *ψ*_1_ being either 0 or $t_{cdf}^{-1}\left(1-\alpha ;N-rank\left(M\right)\right)/\sqrt{N}$, where *α* = 0.05 is the significance level of the permutation test to be performed at a later stage, ensuring a calibrated signal strength sufficient to yield an approximate power of 50% with Gaussian errors, irrespective of the sample size. The actual effect was coded in the first regressor only, here denoted **m**, the second regressor modelling an intercept. This regressor was constructed as a set of random values following a Gaussian distribution with zero mean and unit variance. As in real experiments, such effects of interest may be (as with the residuals) not independent across observations, three different values for the strength of this dependence were simulated, using *h*_**m**_^2^ = {0, 0.4, 0.8} These values are equivalent to the heritability of **m** in the context of genetics, yet without loss of generality to studies in which there is dependence between the data that constitute any individual independent variable, including certain designs involving repeated measurements.^4^

Permutations, sign flippings, and permutations with sign flippings were performed, either freely or respecting the dependence structure. In each case, 500 shufflings were performed for each of the 500 variables, and the whole process was repeated 500 times, allowing histograms of p-values to be constructed, as well as to estimate the variability around the heights of the histogram bars. Confidence intervals (95%) were computed for the empirical error rates and power using the Wilson method (Wilson, 1927). Significance levels were also compared using Bland–Altman plots (Bland and Altman, 1986), modified so as to include the confidence intervals around the means of the methods. The histograms and Bland–Altman plots are shown in the Supplementary Material.

### Power

**Fig. 6:**
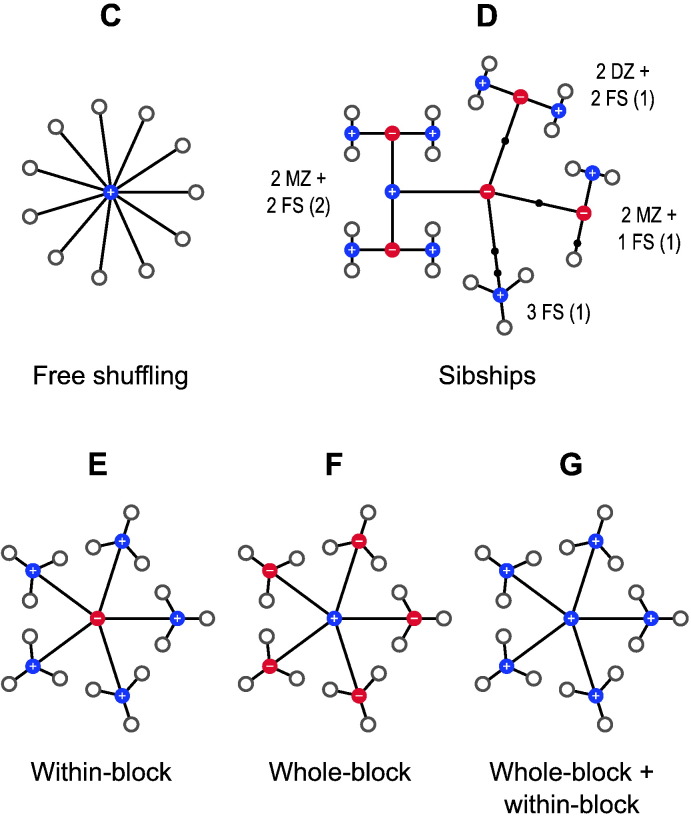
Tree diagrams c–g, used to assess power, in addition to a, b, h and i (shown in Fig. 5, Fig. 7, Fig. 8). In c, observations can be shuffled without restrictions. In d, which represent a set of five sibships, mz refers to each subject of a pair of monozygotic twins, dz to dizygotic twins, and fs to full siblings (non-twin and not half siblings); the numbers in parentheses indicate the number of each type of sibship in the tree (see also Fig. 7). In e, observations can be shuffled only within-block; in f the blocks as a whole can be shuffled, and in g, shufflings are allowed within-block, and the blocks as a whole can also be shuffled.

**Fig. 7:**
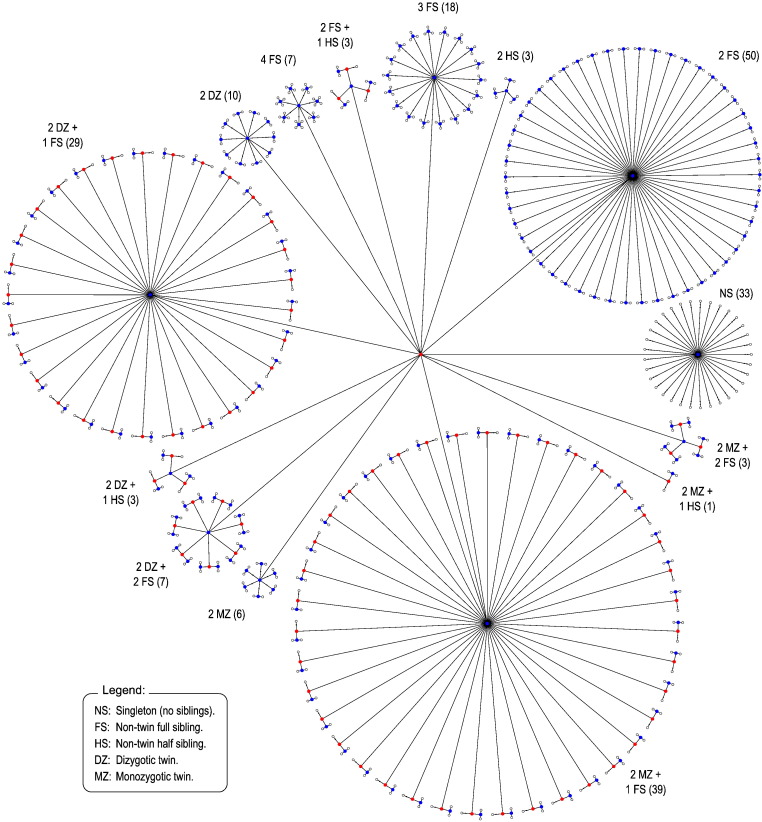
Tree diagram depicting the structure present among the subjects of the Human Connectome Project hcp, at the time of the release hcp-s500, with 518 subjects. The numbers in parentheses indicate how many of each type of sibship set are present.

**Fig. 8:**
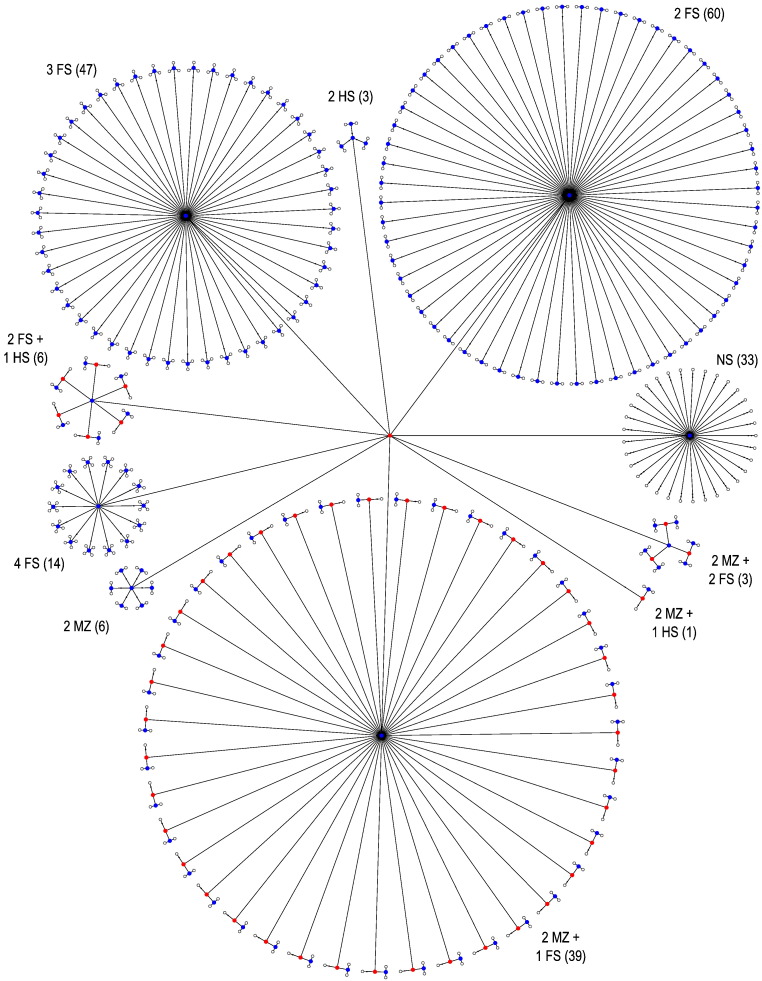
Tree diagram representing the structure among the same 518 subjects of the hcp-s500 release, shown in Fig. 7, but treating dizygotic twins as ordinary siblings, therefore not accounting for the possibility of shared common non-genetic effects within dizygotic twin pair.

The evaluations above were used to assess error rates and power according to the degree of non-independence between observations and distribution of the errors. To further investigate how the restrictions imposed by the exchangeability blocks could affect power, other dependence structures were considered to shuffle the data, in addition to the datasets a and b above; these were named c through i (Fig. 6, Fig. 7, Fig. 8). The configuration c corresponds to freely shuffling 11 observations; d corresponds to a small set of 5 sibships with a total of 18 subjects, mixing whole-block and within-block at different levels; e is formed by 15 observations, organised in 5 blocks of 3 observations each, with shufflings being allowed within-block only; f is similar, but with whole-block rearrangements only, and g also similar, but allowing both whole-block and within-block simultaneously; configurations h and i use the family structure of the Human Connectome Project at the time of the hcp-s500 release (more details below): in h, dizygotic twins are treated as a category on its own, thus accounting for the possibility of shared, non-genetic effects within twin pair, whereas in i, dizygotic twins are treated as ordinary, non-twin full siblings. The number of possible permutations and sign flippings for each of these structures is shown in Table 1.

For each of these nine datasets, an artificial effect (signal) was introduced, in the same way as described in the previous section, but here exclusively using independent Gaussian errors and preserving this independence throughout the simulations while still using multi-level exchangeability blocks for shuffling, as if dependencies among the data existed. Power was then compared with what would be observed if the same data were shuffled without the restrictions imposed by the ebs. For each configuration, 100 repetitions were performed, each simulating 1000 variables (as before, each could represent a voxel or vertex in an image). Up to 512 shufflings were used, either permutations, sign flippings, or permutations with sign flippings. Each repetition used a different set of random observations and a different set of shufflings when the maximum number of possible rearrangements was larger than the number of shufflings performed. The significance level was set as α=116=0.0625. Both the number of permutations and the significance level were chosen so as to allow compatible resolutions of the p-values among runs, allowing a more direct comparison between each case.

Power changes were assessed in relation to what would be observed if the data were shuffled freely, and compared to a measure of the amount of shuffling applied to the data, given the restrictions imposed by the permutation tree. For this purpose, the Hamming distance (Hamming, 1950) was used; this distance counts the number of observations that change their position at each permutation (ee) or that change their sign at each sign flip (ise), or both when permutations are performed together with sign flippings. While the Hamming distance cannot be interpreted as a direct quantification of perturbation on the data, it is appropriate to quantify the effect of the shufflings proper, which do not depend on actual data values.

### Real data

The ongoing Human Connectome Project (hcp) involves the assessment of about four hundred sibships, in many cases with up to four subjects, and with at least one pair of monozygotic (mz) or dizygotic (dz) twins (Van Essen et al., 2012, Van Essen et al., 2013). The inclusion of additional siblings to the classical twin design is known to improve the power to detect sources of variation in the observable traits (Posthuma and Boomsma, 2000, Keller et al., 2010). The objective is to have a genetically informative sample as in a classical twin design, enriched with the inclusion of relatives from the same nuclear family. The coefficient of kinship between mz twins is the same for all such pairs, and so are their expected covariance. Likewise, the covariance is the same for all pairs of dz twins. While kinship can be modelled, such modelling is contingent upon various assumptions that may not always be valid, or that can be hardly checked for all the imaging modalities and exploratory analyses that the hcp entails. Instead, such dependence structure can be represented as a tree that indicates which pieces of data can be shuffled for inference, rendering the permutation methods described this far directly applicable to the hcp data, and without the need to explicitly model the exact degree of dependence present in the data. Depending on whether there is interest in considering or not common effects in dizygotic twins, these can be treated as a category on their own, that cannot be shuffled with ordinary, non-twin siblings, or be allowed to be shuffled with them (Fig. 7, Fig. 8).

Virtually all data being collected in the hcp are to be publicly released,^5^ and at the time of this writing, the available data are the hcp-s500, which includes various imaging and non-imaging measurements for approximately five hundred subjects. Here, measurements of height, weight, and body mass index (bmi) (Barch et al., 2013) were investigated for positive and negative associations with the cortical area and thickness as measured at each point of the cortex. These traits are well known to be highly heritable, with most studies reporting *h*^2^ estimates that are well above 0.70, so that measurements on subjects from the same family cannot be considered independent. [For the heritabilities of height, weight and bmi, see Farooqi and O'Rahilly, 2005, Visscher et al., 2006, Walley et al., 2006, Silventoinen and Kaprio, 2009, Silventoinen et al., 2012, Min et al., 2013; for cortical thickness and area, see Panizzon et al., 2009, Winkler et al., 2010, Joshi et al., 2011, Eyler et al., 2011, Eyler et al., 2012, Kremen et al., 2013, McKay et al., 2014, among others.] To confirm the heritability of these traits specifically in the hcp sample, the variance of these traits was decomposed into genetic and environmental components using the maximum-likelihood methods described in Almasy and Blangero (1998), and as implemented in the package Sequential Oligogenic Linkage Analysis Routines — solar (Department of Genetics, Texas Biomedical Research Institute, San Antonio, TX, usa). The released hcp data do not include an index that could directly categorise subjects according to a common environment or household. Nonetheless, ignoring these possible effects has the potential to overestimate heritabilities. To minimise this possibility, two models were tested: one in which a common environment term (*c*^2^) was not included, and another in which a rather conservative surrogate for household effects was included; such proxy was defined by assigning all subjects sharing the same mother to a common environment. The reasoning is twofold: to account for potential maternal effects, which could affect half-siblings sharing the same mother, but not those sharing the same father, and also considering that, most commonly, children of divorced couples tend to stay or dwell with their mothers for most of the time. To ensure normality, the traits were subjected to a rank-based inverse-normal transformation before estimation. The nuisance variables included in the model were age, age-squared, race and ethnicity, the interactions of these with sex, as well as sex itself. The test statistic, for either *h*^2^ and *c*^2^, is twice the difference between the log-likelihood of a model in which the parameter being tested is constrained to zero and the log-likelihood of a model in which that parameter is allowed to vary; this statistic (deviance) is distributed as 50:50 mixture of a point mass and a *χ*^2^ distribution with one degree of freedom (Self and Liang, 1987); here we call this statistic 2*D*_LL_. For this analysis, 502 subjects with complete data for all these variables were selected (mean age: 29.22, standard deviation: 3.47, range 22–36 years; 296 females; 49 mz pairs, 356 non-mz sibling pairs, 16 half-sibling pairs).

The imaging protocol used for the structural magnetic resonance scans, as well as the steps necessary to construct the surface representation of the cortical mantle, have been described extensively in Glasser et al. (2013) (see also the references therein); FreeSurfer (Martinos Center for Biomedical Imaging, Massachusetts General Hospital, Boston, ma, usa) was used to generate the surfaces and to obtain cortical thickness measurements (Dale et al., 1999, Fischl et al., 1999, Fischl and Dale, 2000); image registration was performed using the Multimodal Surface Matching (msm) framework (Robinson et al., 2014). The surface area was processed using the methods described in Winkler et al. (2012): the area was measured at each face of the white surface, then interpolated using a pycnophylactic method to a common grid (an icosahedron recursively subdivided five times, therefore with 10,242 vertices and 20,480 faces), and finally converted from facewise to vertexwise. Cortical thickness was also resampled to the same resolution, using barycentric interpolation. Both thickness and area were smoothed on the surface of a homeomorphic sphere with 100 mm radius using a Gaussian kernel with full width at half maximum (fwhm) of 20 mm. For these analyses, 5000 permutations were used, and dz twins were considered as constituting a category on their own, and therefore not allowed to be permuted with non-twin siblings in the same family. Nuisance variables were the same used for the heritability analyses described above, plus global cortical surface area and average thickness. Visualisation of imaging results used Blender (The Blender Foundation, Amsterdam, The Netherlands). Sample statistics for the analysed traits are shown in Table 2.

## Results

### Error rates and power

Despite the differences in the relationship between the observations that constituted datasets a and b, the results were very similar. With errors that were independent and symmetric, i.e., either normally distributed (Gaussian) or kurtotic (Laplacian), the false positive rates (error type i) were controlled at the nominal level (*α* = 0.05) using unrestricted permutations, sign flippings, or permutations with sign flippings, whenever there were no true dependence between observations or elements of the regressor of interest, that is, when either *h*_**ϵ**_^2^ or *h*_**m**_^2^ was equal to zero. With both *h*_**ϵ**_^2^ and *h*_**m**_^2^ higher than zero, however, the conventional test in which the data are shuffled freely became, as expected, invalid. Using instead the shuffling strategy that we propose, that respects the covariance structure present or assumed to exist in the data, the false positive rates were controlled at α, even when the dependence was at high levels. These results are shown in Table 3 (Gaussian) and in the Supplementary Table 1 (Laplacian).

With skewed (Weibullian) errors, sign flippings were generally conservative when *h*_**ϵ**_^2^ or *h*_**m**_^2^ were equal to zero and the data were shuffled freely. With *h*_**ϵ**_^2^ and *h*_**m**_^2^ higher than zero, the test not only reversed its conservativeness, but became invalid if flippings ignored the data structure. If, however, the shufflings were performed respecting the restrictions imposed by the relationships among the datapoints, the test was valid in all cases, with its conservativeness maintained. These results are shown in the Supplementary Table 2.

These tables also show the power of each shuffling strategy when there is true signal present. For the cases in which the false positive rate is not controlled, the test is invalid, and as a consequence, considerations of power are irrelevant; in these cases, the values that would represent power are shown crossed by a line. When the data are truly independent, hence unrestricted shuffling could be performed, the proposed restricted permutations caused a slight, yet consistent, loss of power for the datasets a and b. This is revisited in the next section, with the other synthetic datasets.^6^

### Power

For the nine synthetic datasets, a slight, yet consistent loss of power was observed when using the proposed restricted shuffling strategy, compared to the results using unrestricted shuffling when the last was, in fact, possible. These results are shown in Table 4. The loss appears to be larger for the datasets with more involved dependence structures (e.g., dataset d), or when restrictions on permutations are imposed at higher levels (e.g., dataset e), or on sign flippings at lower levels (e.g., datasets f and g). Even so, this is not quite as conspicuous with samples that are just modestly larger (e.g., a and b), or much larger (e.g., datasets h and i, that use the data structure from the hcp).

**Fig. 9:**
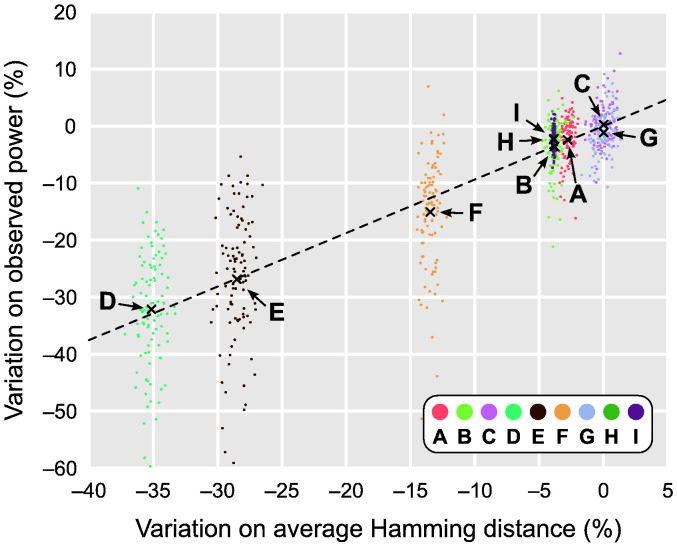
Changes in power related well to the average Hamming distance across permutations for the nine simulated datasets a–i (see also Table 4). When all dots are considered, *R*^2^ = 0.7557 for a linear fit (dashed line); when only the centres of mass for each dataset (marked with “×” and indicated with arrows) are considered, *R*^2^ = 0.9902.

With exchangeable errors, in which only permutations are performed, power reductions were more noticeable for some datasets and related well to how the data could be disarranged at each permutation, as quantified by the average Hamming distance across the permutations that were performed. This is shown in Table 4, and also visually in Fig. 9. With independent and symmetric errors, in which only sign flippings are performed, the power losses were considerably smaller, and unrelated to the Hamming distance. In the same manner, permutations combined with sign flippings showed power changes that were minimal, and unrelated to the Hamming distance. Moreover, in these cases the resulting power was, for all datasets, higher than for just permutations or just sign flippings.

Table 4 and Fig. 9 show a considerable dispersion of the observed power around the average. In the simulations, this dispersion can be reduced by one order of magnitude approximately just by using the same data and design for all repetitions, varying only the set of shufflings that are performed. Although this reduced dispersion would reflect more accurately the actual variation that different shufflings would cause on a given real experiment, the average power would be dependent on the exact, but random values used for the simulations, and would not be appropriate for the investigation performed here. The magnitude of variations on power as shown does not translate to actual experiments and should not be interpreted as such.

### Real data

Summary statistics for height, weight, bmi, global cortical surface area, and global average thickness for the analysed hcp sample are shown in Table 5. The same Table also shows that, consistently with the literature, all these quantities are highly and significantly heritable, even when a conservative surrogate for common environment is included in the model. In fact, the estimated common environment fraction of the variance (*c*^2^) was zero for all traits except for height. When the shared environment term is removed from the model, the estimated heritability for height increases to 0.8771 (standard error: 0.0244, 2*D*_LL_ = 146.9, p-value: 4.1 · 10^− 34^).

**Fig. 10:**
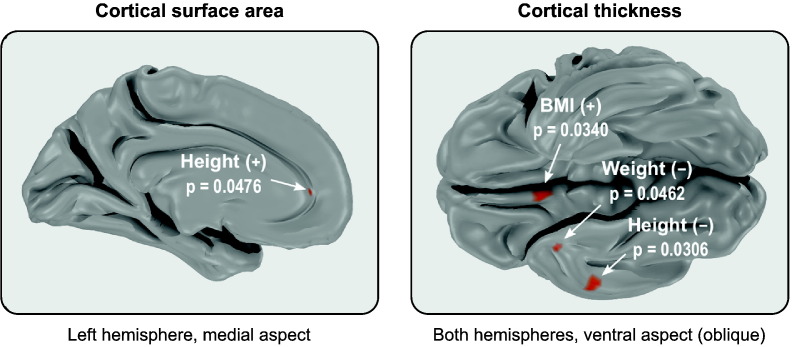
Maps showing the locations of the peaks of significance, for positive (+) and negative (−) correlations of height, weight, and bmi with cortical surface area and thickness. For conciseness, and given their lack of overlap, the original maps for thickness were thresholded at 0.05 and added together, allowing the regions to be displayed in the same figure. Even after using fwer-correction across the brain and contrasts, the unrestricted shuffling identified seemingly significant regions; these regions were not found significant using the restricted permutations that respect the family structure in the hcp sample. Provided that these traits are highly non-independent between subjects (i.e., heritable) this suggests that these results, produced with simple, unrestricted permutation, are in fact **false positives** (the peaks of significance for both restricted and unrestricted are listed in Supplementary Table 3).

Permuting the data freely to test the hypotheses of correlation between thickness or area and indices of body size, therefore not respecting the structure of the sibships, allowed the identification of a few seemingly significant associations, even after fwer correction across the whole brain, and considering that both positive and negative tests were being performed. These regions, shown in Fig. 10, are (1) the left anterior cingulate for a positive correlation between height and cortical surface area, (2) the right orbitofrontal medial cortex for a positive correlation between thickness and bmi, (3), the right temporal pole, at the confluence of the inferior temporal gyrus, for a negative correlation between thickness and body weight, and (4) the right inferior temporal gyrus for a negative correlation between thickness and height. All these regions are very small, two of them comprising just one vertex at the resolution of the surfaces. However, using the proposed multi-level permutation strategy, in which shufflings only happen within siblings of the same type, and in which families with identical structure are allowed to be permuted as a whole, therefore respecting the kinship structure, all these findings became no longer significant. Supplementary Table 3 shows the minimum (most significant) p-value throughout the brain for both unrestricted and restricted permutation.

## Discussion

### Error rates and power

The proposed multi-level shuffling strategy controlled the false positive rate at the nominal level in all configurations evaluated. With the only exception of sign flippings in the presence of skewed errors, which clearly violates assumptions, the empirical distribution of p-values was uniform, as desired, whenever shufflings respected the dependence structure present in the data; ignoring the dependence resulted in inflated error rates, rendering the test invalid. Having dependence in both data and model may seem unusual, but in fact, this is a quite common scenario, as exemplified with the data from the hcp.

The guaranteed validity and exactness of p-values came, however, at the price of a small, yet noticeable and consistent reduction in power, that related to the complexity of the dependence structure and the ensuing restrictions on exchangeability. This can be understood by noting that the restricted permutation strategy does not disarrange the data as much as the unrestricted shuffling, with the consequence that the statistics computed after permuting the data may not be as distant from the statistic computed from the unpermuted data. With sign flippings, the power losses were smaller, and unrelated to the Hamming distance, presumably because even changes seemingly small, such as a single sign swap, can cause large perturbations on the shuffled data that are sufficient to minimise reductions on sensitivity. Permutations combined with sign flippings showed minimal power changes that were also unrelated to the average Hamming distance, and with losses that were smaller than for just permutations or just sign flippings, suggesting that when both ee and ise are valid for a given model, permutations with sign flippings can allow maximum efficiency.^7^

Although the diminished sensitivity could suggest that the multi-level permutation strategy would be “conservative”, this is not the case, as can be attested by the exact control over error rates. This apparent incongruity can be understood through the Bland–Altman plots shown in the Supplementary Material, that show that the differences in uncorrected p-values between both strategies is largely outside the margins of the confidence interval in both directions, suggesting that, under the null, variations in p-values can go in any direction when the strategies are compared. Nonetheless, in the presence of signal, or when the p-values are corrected for multiple testing using the distribution of the largest statistic across variables (such as voxels), the p-values for the restricted strategy tend to be stochastically larger than those for the free shuffling.

The restrictions imposed on the possible rearrangements that can be performed, with the consequent reduction in the number of possible permutations, as well as the lessened sensitivity, could be seen as undesirable, but in fact, such restrictions establish a set of rules under which permutation inference can be performed in settings where otherwise it would not possible without appealing to often untenable assumptions, or that would not be possible at all. Simple permutation, if performed, would create data that could be impossible to be observed in practice, and thus, that should not be used to construct the reference distribution to test the hypotheses of interest. Moreover, the stronger the dependency is between observations, the fewer genuinely independent pieces of information are available to test a given hypothesis; in this scenario, smaller power does not appear unexpected.

### Body size and cortical morphology

Height, weight, and bmi are known to be highly heritable in general, and were so for the hcp sample. Likewise, the heritability for global cortical surface area and average thickness are known to be heritable, and were found as such in the sample analysed. All these traits remained highly heritable even when a potential confound — a surrogate for household and maternal effects — was included. Even if estimated heritability were reduced, common effects would still cause the observations not to be independent. The fact that for all these traits there is a strong dependence between the observations implies that a permutation test that ignores the relationship between observations would not be valid, by violating exchangeability.

Indeed, the test that shuffled the data freely identified a few positive and negative localised significant associations between indices of body size and cortical area and thickness, even after fwer correction considering all tests in both hemispheres and the fact that positive and negative hypotheses were being tested. None of these areas were found to be significant if the test used only permutations that respected the structure present in the data, in the multi-level fashion, suggesting that these findings are likely false positives. None of the regions implicated were reported in previous studies that investigated relationships between indices of body size and cortical morphology (Pannacciulli et al., 2006, Raji et al., 2010, Ho et al., 2010, Ho et al., 2011, Smucny et al., 2012, Curran et al., 2013, Marqués-Iturria et al., 2013, Melka et al., 2013, Bond et al., 2014) that we could identify. It should be conceded, however, that not all these studies used the same methods, with some having analysed grey matter volumes in voxel-based representations of the brain, and some, despite using surface-based methods, performed analyses in macroscopic regions, as opposed to at each point in the cortical mesh. Still, as the simulations demonstrated, the violation of the exchangeability assumption makes the free permutation prone to inflated amounts of error type i if the observations are not independent, and the absence of similar findings from the literature supports the likelihood that these seemingly significant findings are not genuine, being instead false positives.^8^

Another aspect is that, although fwer-correction was applied considering the multiplicity of vertices in the mesh representation of the cortex and the two contrasts (positive and negative), no correction was applied considering that overall six tests were performed (three independent variables versus two dependent variables); fwer-controlling procedures that would take into account the non-independence between these tests are currently not available. Using Bonferroni correction, the results using the free permutation, which are likely false-positives as discussed above, disappear. Since most studies — and in fact, most of those referenced in the previous paragraph — investigated only the relationship between one independent versus one dependent variable, for which no such correction is necessary, the results shown emulate well the risk of false positives in similar, real studies.

### Applications and other considerations

In addition to the above examples, and most clearly, with a direct example application for the hcp data, the multi-level permutation strategy can be considered for repeated measurements designs in which within- and between-subject factors are tested in the same model or contrast, such as for a continuous regressor. A direct comparison of the power observed for datasets e, f and g, using permutations only, shows that even with the same number of subjects, the combination of within-block with whole-block permutation can be more powerful than each of these used in isolation. Moreover, the strategy can also be considered when not all measurements for a given subject are available, as long as compound symmetry within subject remains valid, without the need to exclude entirely the data for subjects as would be the case for whole-block permutation.

As experiments are planned considering strategies for subsequent analyses, the use of permutation tests can be included among the tools available, especially given its simplicity and, as demonstrated here and in a large body of literature, flexibility to accommodate designs and data that can be quite complex. Adequate planning includes ensuring that assumptions for permutation tests are met from the beginning, such as that the random errors of the instrument are stable along time, and do not vary with the values they measure, that the observations if not independent, possess a dependence structure that can be accommodated in a framework as the one shown here, and that observations, if not homogeneous, can be broadly qualified into a few number of variance groups.

Indeed, regarding vgs, the compatibility of these with the blocks ensures the feasibility of permutation tests, but it also allows that the necessary assumptions are reduced to a minimum: instead of requiring that all observations are homoscedastic (strong), the maximum possible amount of heterogeneity of variances that could still permit the shuffling as indicated by the blocks (weaker assumption) can be allowed. In this case, homogeneity would still be there, although not across all and every observation, just the minimal amount necessary so that the experiment can still be analysed. These considerations may not be relevant if the recruiting process, experimental conditions or data collection can guarantee that the same variance is homogeneous, but may be necessary when the data collection or samples are not under direct control of the researcher (e.g., reanalysis of past experiments, or census data).

## Conclusion

The proposed multi-level block permutation effectively controls the false positive rate, even in the presence of strong dependence between observations, and can be used as a general inference tool when the dependence structure can be organised in blocks, hierarchically if necessary. There is an unavoidable loss of power due to reduced scope of shuffling, although in large datasets, with relatively complex dependence structure, such as the hcp, this loss is expected to be quite small, especially if permutations can be combined with sign flippings.

## Figures and Tables

**Table 1:** Number of permutations (ee) and sign flippings (ise) for the 9 dependence structures simulated to examine power. If there were ties in the data, the number of possible permutations would be smaller. When both ee and ise can be used, such that the data can be permuted and sign flipped, the number of possible rearrangements is simply the product of the number of permutations with the number of sign flippings. The footnote shows in detail how these values were calculated for the more complex configurations.

	Unrestricted shuffling		Restricted shuffling	
Set	ee	ise	ee	ise
a	36! ≈ 3.7 ⋅ 10^41^	2^36^ ≈ 6.9 ⋅ 10^10^	4^9^ ⋅ 9! ≈ 9.5 ⋅ 10^10^	2^9^ = 512
b	27! ≈ 1.1 ⋅ 10^28^	2^27^ ≈ 1.3 ⋅ 10^8^	2^9^ ⋅ 9! ≈ 1.9 ⋅ 10^8^	2^9^ = 512
c	11! ≈ 4.0 ⋅ 10^7^	2^11^ = 2048	11! ≈ 4.0 ⋅ 10^7^	2^11^ = 2048
d	18! ≈ 6.4 ⋅ 10^15^	2^18^ = 262144	2^8^ ⋅ 3! = 1536	2^5^ = 32
e	15! ≈ 1.3 ⋅ 10^12^	2^15^ = 32768	(3!)^5^ = 7776	2^15^ = 32768
f	15! ≈ 1.3 ⋅ 10^12^	2^15^ = 32768	5! = 120	2^5^ = 32
g	15! ≈ 1.3 ⋅ 10^12^	2^15^ = 32768	(3!)^5^ ⋅ 5! = 933120	2^5^ = 32
h	518! ≈ 6.5 ⋅ 10^1182^	2^518^ ≈ 8.6 ⋅ 10^155^	*a* ≈ 2.9 ⋅ 10^287^	*c* ≈ 6.6 ⋅ 10^63^
i	518! ≈ 6.5 ⋅ 10^1182^	2^518^ ≈ 8.6 ⋅ 10^155^	*b* ≈ 1.3 ⋅ 10^335^	*d* ≈ 6.6 ⋅ 10^63^

*a* = [33!] ⋅ [2^50^ ⋅ 50!] ⋅ [2^3^ ⋅ 3!] ⋅ [(3!)^18^ ⋅ 18!] ⋅ [2^3^ ⋅ 3!] ⋅ [(4!)^7^ ⋅ 7!] ⋅ [2^10^ ⋅ 10!] ⋅ [2^29^ ⋅ 29!] ⋅ [2^3^ ⋅ 3!] ⋅ [(2^2^)^7^ ⋅ 7!] ⋅ [2^6^ ⋅ 6!] ⋅ [2^39^ ⋅ 39!] ⋅ [2] ⋅ [(2^2^)^3^ ⋅ 3!].

*b* = [33!] ⋅ [2^60^ ⋅ 60!] ⋅ [2^3^ ⋅ 3!] ⋅ [(3!)^47^ ⋅ 47!] ⋅ [2^6^ ⋅ 6!] ⋅ [(4!)^14^ ⋅ 14!] ⋅ [2^6^ ⋅ 6!] ⋅ [2^39^ ⋅ 39!] ⋅ [2] ⋅ [(2^2^)^3^ ⋅ 3 !].

*c* = 2^33^ ⋅ 2^50^ ⋅ 2^3^ ⋅ 2^18^ ⋅ 2^3^ ⋅ 2^7^ ⋅ 2^10^ ⋅ 2^29^ ⋅ 2^3^ ⋅ 2^7^ ⋅ 2^6^ ⋅ 2^39^ ⋅ 2 ⋅ 2^3^ = 2^212^.

*d* = 2^33^ ⋅ 2^60^ ⋅ 2^3^ ⋅ 2^47^ ⋅ 2^6^ ⋅ 2^14^ ⋅ 2^6^ ⋅ 2^39^ ⋅ 2 ⋅ 2^3^ = 2^212^.

Compare the products *a*, *b*, *c*, and *d* with Fig. 7, Fig. 8, that depict respectively the hcp structures h and i; the factors are shown starting from the singletons (labelled as ns in the figures) and running counter-clockwise around the central node.

**Table 2:** Descriptive statistics for the indices of body size and for global cortical surface area and global average thickness on the sample of subjects from the hcp.

Trait	Mean ± sd	Range
Height (m)	1.708 ± 0.096	1.473–1.956
Weight (kg)	77.712 ± 17.342	44.906–128.820
bmi (kg/m^2^)	26.581 ± 5.252	16.788–45.171
Area (cm^2^)	1666.80 ± 169.79	1292.14–2112.00
Thickness (mm)	2.620 ± 0.087	2.239–2.824

**Table 3:** Proportion of error type i and power (%) for the simulated sets a and b, with Gaussian errors, at the level *α* = 0.05, using different degrees of dependence for the error terms (*h*_**ϵ**_^2^) and for the regressor of interest (*h*_**m**_^2^), using permutations (ee), sign flippings (ise), or permutations with sign flippings (ee + ise). Confidence intervals (95%) are shown between parentheses. The values that appear striked out are not valid, as they refer to power observed when the corresponding error rates are not controlled (i.e., the lower bound of the confidence interval is above the nominal level *α* when there is no actual effect). For the Laplace and Weibull errors, please see the Supplementary Material.

Set	*h*_**ϵ**_^2^	Unrestricted shuffling						Restricted shuffling					
Without effect (error rate)			With effect (power)			Without effect (error rate)			With effect (power)		
*h*_**m**_^2^ = 0	*h*_**m**_^2^ = 0.4	*h*_**m**_^2^ = 0.8	*h*_**m**_^2^ = 0	*h*_**m**_^2^ = 0.4	*h*_**m**_^2^ = 0.8	*h*_**m**_^2^ = 0	*h*_**m**_^2^ = 0.4	*h*_**m**_^2^ = 0.8	*h*_**m**_^2^ = 0	*h*_**m**_^2^ = 0.4	*h*_**m**_^2^ = 0.8
*Permutations only:*													
a	0.0	5.0 (3.4–7.3)	4.9 (3.3–7.2)	5.1 (3.5–7.3)	49.1 (44.7–53.5)	47.4 (43.1–51.8)	46.5 (42.2–50.9)	5.0 (3.4–7.3)	4.9 (3.3–7.2)	5.1 (3.5–7.4)	47.6 (43.3–52.0)	46.1 (41.7–50.5)	44.3 (40.0–48.7)
	0.4	5.0 (3.4–7.3)	6.4 (4.5–8.9)	7.8 (5.7–10.5)	49.8 (45.4–54.2)	49.8 (45.4–54.1)	48.5 (44.2–52.9)	5.1 (3.5–7.3)	5.0 (3.4–7.3)	5.0 (3.4–7.3)	48.5 (44.1–52.9)	44.1 (39.8–48.5)	38.6 (34.4–42.9)
	0.8	4.9 (3.4–7.2)	7.8 (5.8–10.5)	10.4 (8.0–13.3)	51.5 (47.1–55.8)	50.5 (46.2–54.9)	49.7 (45.3–54.1)	4.9 (3.3–7.1)	5.0 (3.4–7.3)	5.0 (3.4–7.3)	50.6 (46.2–54.9)	41.9 (37.6–46.2)	34.3 (30.2–38.5)
b	0.0	5.0 (3.4–7.2)	5.0 (3.4–7.2)	4.9 (3.4–7.2)	48.4 (44.1–52.8)	47.4 (43.0–51.7)	46.5 (42.1–50.8)	4.9 (3.3–7.2)	5.0 (3.4–7.3)	5.0 (3.4–7.3)	46.7 (42.4–51.1)	45.4 (41.1–49.8)	43.8 (39.5–48.2)
	0.4	5.0 (3.4–7.3)	6.2 (4.4–8.7)	7.6 (5.6–10.2)	49.6 (45.2–53.9)	49.2 (44.8–53.6)	48.2 (43.9–52.6)	5.0 (3.4–7.2)	5.0 (3.4–7.3)	5.0 (3.4–7.3)	48.3 (43.9–52.7)	43.5 (39.2–47.8)	38.1 (34.0–42.5)
	0.8	5.0 (3.4–7.3)	7.4 (5.4–10.0)	10.0 (7.7–12.9)	50.4 (46.0–54.8)	50.3 (45.9–54.6)	49.2 (44.8–53.6)	5.0 (3.4–7.3)	4.9 (3.3–7.2)	5.0 (3.4–7.3)	50.0 (45.6–54.4)	41.7 (37.5–46.1)	33.7 (29.7–37.9)

*Sign flippings only:*													
a	0.0	5.1 (3.5–7.4)	5.0 (3.4–7.3)	4.9 (3.3–7.2)	45.6 (41.3–50.0)	45.6 (41.3–50.0)	45.1 (40.8–49.5)	5.0 (3.4–7.3)	5.1 (3.5–7.4)	5.2 (3.6–7.5)	41.5 (37.2–45.9)	41.7 (37.5–46.1)	40.9 (36.7–45.3)
	0.4	5.0 (3.4–7.2)	6.2 (4.4–8.6)	7.7 (5.7–10.4)	47.3 (42.9–51.6)	47.1 (42.7–51.5)	46.0 (41.7–50.4)	4.9 (3.3–7.1)	5.0 (3.4–7.3)	5.2 (3.6–7.5)	43.0 (38.8–47.4)	39.2 (35.0–43.6)	34.8 (30.8–39.1)
	0.8	5.1 (3.5–7.4)	7.6 (5.6–10.3)	10.7 (8.3–13.7)	48.5 (44.1–52.9)	48.3 (43.9–52.7)	48.6 (44.2–52.9)	4.9 (3.3–7.1)	5.0 (3.4–7.3)	5.2 (3.6–7.5)	45.2 (40.8–49.5)	37.6 (33.5–41.9)	31.6 (27.7–35.9)
b	0.0	5.0 (3.4–7.2)	4.9 (3.4–7.2)	5.0 (3.4–7.2)	45.1 (40.8–49.5)	44.3 (40.0–48.7)	43.8 (39.6–48.2)	4.9 (3.4–7.2)	5.1 (3.5–7.4)	5.3 (3.6–7.6)	41.5 (37.3–45.9)	40.9 (36.7–45.3)	39.9 (35.7–44.2)
	0.4	5.0 (3.4–7.3)	6.3 (4.4–8.7)	7.4 (5.4–10.0)	46.3 (42.0–50.7)	45.3 (41.0–49.7)	46.2 (41.9–50.6)	4.9 (3.3–7.2)	5.1 (3.5–7.4)	5.1 (3.5–7.4)	42.4 (38.2–46.8)	38.3 (34.2–42.7)	35.2 (31.2–39.5)
	0.8	5.1 (3.5–7.3)	7.6 (5.6–10.3)	10.1 (7.8–13.1)	47.5 (43.2–51.9)	47.2 (42.8–51.5)	47.1 (42.8–51.5)	4.8 (3.2–7.0)	5.0 (3.4–7.3)	5.0 (3.4–7.3)	44.2 (39.9–48.6)	37.3 (33.2–41.6)	30.8 (26.9–34.9)

*Permutations + sign flippings:*													
a	0.0	5.1 (3.5–7.4)	5.0 (3.4–7.2)	5.0 (3.4–7.2)	48.6 (44.3–53.0)	48.6 (44.3–53.0)	46.8 (42.5–51.2)	5.1 (3.5–7.4)	5.1 (3.5–7.4)	5.3 (3.6–7.6)	48.4 (44.0–52.7)	48.8 (44.4–53.1)	46.9 (42.6–51.3)
	0.4	5.0 (3.4–7.3)	6.4 (4.6–8.9)	7.7 (5.7–10.4)	49.7 (45.3–54.0)	48.6 (44.2–52.9)	48.0 (43.6–52.4)	5.0 (3.4–7.2)	5.1 (3.5–7.4)	5.4 (3.7–7.7)	49.7 (45.4–54.1)	44.5 (40.2–48.9)	40.2 (36.0–44.6)
	0.8	5.0 (3.4–7.2)	7.7 (5.7–10.4)	10.5 (8.1–13.5)	51.3 (46.9–55.6)	50.3 (45.9–54.6)	50.2 (45.8–54.5)	4.9 (3.3–7.2)	5.1 (3.5–7.4)	5.4 (3.7–7.7)	52.1 (47.7–56.5)	43.2 (38.9–47.6)	36.3 (32.2–40.6)
b	0.0	5.1 (3.5–7.4)	5.1 (3.5–7.3)	5.0 (3.4–7.2)	48.8 (44.4–53.1)	48.2 (43.8–52.5)	47.1 (42.8–51.5)	5.1 (3.5–7.4)	5.2 (3.6–7.5)	5.3 (3.7–7.7)	48.3 (44.0–52.7)	48.1 (43.7–52.4)	47.0 (42.7–51.4)
	0.4	5.1 (3.5–7.4)	6.2 (4.4–8.7)	7.5 (5.5–10.2)	49.2 (44.8–53.5)	49.5 (45.1–53.8)	48.2 (43.9–52.6)	5.0 (3.4–7.3)	5.2 (3.5–7.5)	5.3 (3.7–7.7)	49.0 (44.6–53.4)	45.8 (41.5–50.2)	40.6 (36.4–44.9)
	0.8	5.0 (3.4–7.2)	7.6 (5.6–10.3)	10.2 (7.8–13.2)	50.3 (45.9–54.7)	50.3 (46.0–54.7)	50.0 (45.6–54.4)	5.0 (3.4–7.2)	5.2 (3.5–7.5)	5.2 (3.6–7.5)	51.2 (46.8–55.5)	43.6 (39.3–47.9)	36.6 (32.5–40.9)

**Table 4:** Relationship between the average Hamming distance across shufflings and the observed power (± standard deviation). In general, larger reductions in the Hamming distance when using restricted permutations (ee) caused more noticeable losses in power (see also Fig. 9). The loss did not correlate with the Hamming distance when using sign flippings only (ise) or permutations with sign flippings (ee + ise). In these cases, the power changes were generally minimal.

Set	Unrestricted shuffling		Restricted shuffling	
Hamming distance	Power (%)	Hamming distance	Power (%)
*Permutations only:*				
a	34.929 ± 0.051	49.17 ± 7.18	33.956 ± 0.123	47.97 ± 7.22
b	25.945 ± 0.052	48.45 ± 7.77	24.956 ± 0.104	46.68 ± 7.57
c	9.980 ± 0.041	46.52 ± 10.92	9.981 ± 0.044	46.57 ± 10.73
d	16.965 ± 0.044	48.01 ± 10.93	11.003 ± 0.122	32.48 ± 8.48
e	13.973 ± 0.048	47.57 ± 10.23	9.991 ± 0.106	34.58 ± 8.80
f	13.867 ± 0.084	45.16 ± 10.11	12.000 ± 0.000	38.14 ± 8.69
g	13.972 ± 0.047	47.46 ± 10.18	13.975 ± 0.066	46.93 ± 10.21
h	515.996 ± 0.042	49.59 ± 2.37	496.000 ± 0.307	48.41 ± 2.16
i	515.994 ± 0.043	49.56 ± 2.25	496.063 ± 0.326	48.45 ± 2.23

*Sign flippings only:*				
a	17.959 ± 0.131	48.13 ± 6.81	18.000 ± 0.000	44.74 ± 6.28
b	13.476 ± 0.109	47.23 ± 7.66	13.500 ± 0.000	44.06 ± 7.17
c	5.495 ± 0.062	44.16 ± 10.27	5.489 ± 0.069	44.20 ± 10.07
d	8.715 ± 0.375	42.83 ± 10.97	9.000 ± 0.000	38.59 ± 8.75
e	7.492 ± 0.076	45.04 ± 9.05	7.479 ± 0.074	45.00 ± 9.29
f	7.215 ± 0.332	41.81 ± 10.35	7.500 ± 0.000	38.45 ± 7.99
g	7.292 ± 0.366	42.06 ± 10.22	7.500 ± 0.000	38.45 ± 7.99
h	258.528 ± 0.478	49.48 ± 2.23	258.440 ± 0.900	49.36 ± 2.34
i	258.542 ± 0.505	49.59 ± 2.33	258.525 ± 0.761	49.41 ± 2.33

*Permutations with sign flippings:*				
a	35.432 ± 0.042	50.07 ± 7.19	34.913 ± 0.095	50.00 ± 7.19
b	26.449 ± 0.040	49.72 ± 7.74	25.914 ± 0.092	49.24 ± 7.56
c	10.483 ± 0.041	50.30 ± 10.97	10.471 ± 0.033	50.32 ± 10.91
d	17.465 ± 0.041	49.94 ± 11.11	14.450 ± 0.137	46.29 ± 10.55
e	14.471 ± 0.039	49.87 ± 10.19	12.452 ± 0.088	48.81 ± 10.11
f	14.472 ± 0.039	49.93 ± 10.18	13.470 ± 0.085	47.79 ± 9.85
g	14.474 ± 0.035	49.95 ± 10.15	14.456 ± 0.055	49.05 ± 10.16
h	516.480 ± 0.036	49.68 ± 2.26	505.953 ± 0.569	49.65 ± 2.31
i	516.481 ± 0.038	49.61 ± 2.32	506.072 ± 0.570	49.69 ± 2.25

**Table 5:** Heritabilities (*h*^2^) for the indices of body size and for global cortical surface area and global average thickness on the sample of hcp subjects when a surrogate for common environment effects (*c*^2^) is included in the model. The standard errors (se), the test statistic (2*D*_LL_), and the p-values are also shown. Only for height the common environment effect was estimated to be different than zero. All traits being highly heritable implies that permutation for analysis of their relationship must take the dependence structure into account.

Trait	Additive genetic				Common environment			
*h*^2^	se	2*D*_LL_	p-Value	*c*^2^	se	2*D*_LL_	p-Value
Height	0.7346	0.1035	35.4	1.3 ⋅ 10^− 9^	0.1409	0.0990	1.9	8.7 ⋅ 10^− 2^
Weight	0.7248	0.0580	61.1	2.8 ⋅ 10^− 15^	0.0000	–	–	–
bmi	0.7390	0.0572	62.1	1.6 ⋅ 10^− 15^	0.0000	–	–	–
Area	0.8697	0.0274	125.3	2.2 ⋅ 10^− 29^	0.0000	–	–	–
Thickness	0.8961	0.0232	125.5	1.9 ⋅ 10^− 29^	0.0000	–	–	–

## Appendix A Implementation

### Permutation of the tree branches

A naïve way to select only the permutations that respect the data structure would be to create (randomly or lexicographically) permutation matrices as if the data could be shuffled freely, and then test whether these matrices would respect the configuration of the ebs at the various levels. If yes, the permutation matrix is used, otherwise it is discarded. The problem with this approach is not only that the process of testing can be slow, but also the restrictions imposed by the blocks can reduce the number of possible permutations by various orders of magnitude, implying that for some designs, for each valid permutation that is found, enormous numbers of permutations would need to be discarded, in a very inefficient process.

However, the diagram using a tree to represent the blocks and sub-blocks, and their hierarchical relationship, does not constitute merely a visual resource. The same tree structure can be used to efficiently implement a permutation algorithm that shuffles the branches, even in programming languages that do not offer natively a tree type, such as C or Octave/matlab, as trees can be constructed with pointers, or with generic types, such as cells. When constructing the tree representation, each node must store three pieces of information, which are all updated as the permutations and/or sign flips are performed:

1. A three-column array, to be used under the ee assumption, and modified at each permutation, with as many rows as branches beginning at the node. The first column contains a sequence of integers that represents each of the branches. Branches that have identical structure and contain identical leaves (i.e., repeated rows in the relevant part of the design matrix necessary to test the null hypothesis) receive the same index and so this column can contain repeated values. This sequence is used by the Algorithm “l” (Knuth, 2005), the algorithm that performs lexicographic permutations without repetitions; as the algorithm runs, the rows are permuted as a whole. The second column contains the indices that represent the current permutation in relation to the original sequence, that is, the indices that rearrange the original sequence of branches into the current state after permuting. Reverse-indexing this sequence can reset the permuted branches back to the original, unpermuted state. The third column contains the indices that rearrange the previous state into the current state and, just before running the Algorithm “l” for the next permutation, this column is regenerated as a sequence {1, 2, …, *B*}, with *B* here denoting the number of branches (sub-blocks) in the current level; these are the indices that effectively permute the branches. If branches that begin at the node cannot be permuted (negative indices in the multi-level block notation), the whole array is replaced by some distinct marker that can be tested quickly, such as a not-a-number (NaN) or simply a zero (0).
2. A counter, to be used under the ise assumption. The counter can be in a numeral system of radix 2 (e.g., using −1 and +1 as symbols in lieu of the conventional binary 0 and 1), with as many digits as branches starting at the node, and representing in a direct manner the current state of the sign flippings for each branch.
3. The branches that begin at the node. Each branch is constituted of another tree structure, in a pattern that replicates itself recursively from the top level to the most distal branches.

Once the tree has been constructed, shufflings can be performed exhaustively or, if the number of possible rearrangements is too large, only a subset needs to be performed. To generate any single permutation in lexicographic order, the tree is swept from the top node, proceeding recursively down to the next lower level before moving into the next branch in the same level, skipping the nodes in which the last lexicographic permutation has been reached, and stopping when a single pairwise permutation of branches can be performed. The respective branches are then swapped, all previous nodes already visited are reset back to their original, unpermuted state, and a permutation vector is constructed by concatenating the (now permuted) indices of the observations (leaves), from which the permutation matrix is generated.

For sign flippings, the process is similar: the tree is swept, but instead of computing the next permutation, the counter is incremented. Nodes that have reached the last possible sign flipping (i.e., with all signs reversed) are skipped; when a node can have its counter incremented, the sweeping stops, the counter is incremented, and all previous nodes that had been skipped are reset back to their original state. As the counter uses a numeral system with base 2, the counter itself constitutes a vector of sign flips that can be applied to the branches that begin at that node, and generating the sign flipping matrix is then trivial.

As described, the tree representation allows computing exhaustively and lexicographically all possible rearrangements. However, the tree can also be perturbed randomly, with the branches that begin in all its nodes being permuted and/or sign flipped, a feature useful when performing only a small subset of all possible shufflings.

### Variance groups

The most restrictive set of vgs can also be defined from the same tree structure. The nodes of the tree are swept from the first branch in the top node, proceeding recursively down to the next lower level before moving into the next branch in the same level. At the lowest level, observations (leaves) that can be permuted are assigned to the same variance group; those that cannot are each assigned to a distinct group. At the intermediate nodes, between the top node and the terminal leaves, those in which permutation of the branches is allowed have their corresponding vgs defined for the first branch, then replicated for all remaining branches at that level without the need to visit their lower levels. For the nodes in which permutation of branches is not allowed, each branch has its own set of vgs defined. A counter is passed down and up the levels as each node is visited, being incremented to the next integer every time a new vg is created.

### Availability

Implementation for Octave/matlab of these two algorithms, directly usable with the glm for both imaging and non-imaging data, are available in the tool *Permutation Analysis of Linear Models* (palm), which can be downloaded freely from www.fmrib.ox.ac.uk/fsl. We name the first, that permutes the tree branches, as “palm-Tree”.

## Footnotes

1 A *pivotal* statistic has a sampling distribution that does not depend on unknown parameters.

2 Exchangeability with respect to a subset of all possible permutations is termed *weak exchangeability* (Good, 2005). For conciseness, we will use the solitary term “exchangeability”, while making clear the subsets of permutations for which this is valid.

3 The actual skewness and kurtosis for these two distributions are either fixed or functions of their parameters, and therefore were held constant throughout the simulations. For Weibull, the skewness is (Γ(1 + 3/*k*)*λ*^3^ − 3*μσ*^2^ − *μ*^3^)/*σ*^3^ ≈ 19.58, where *k* is the shape and *λ* the scale parameter. For Laplace, the excess kurtosis is 3.

4 Note that errors on measurements are not part of these simulations, such that parameter estimates remain consistent.

5 Detailed information can be found at http://www.humanconnectome.orgwww.humanconnectome.org.

6 Histograms of p-values using permutations, sign flippings, and permutations with sign flippings, for cases of normal and skewed distributions, and using both unrestricted and restricted shuffling, for dataset a, are shown in Supplementary Fig. 1 (the pattern is similar for dataset b). These extend the results shown in Table 3 and in Supplementary Tables 1 and 2, with an overview of the frequencies of p-values throughout the whole [0 1] interval. Except for the use of the ise with skewed errors (assumptions violated), in general the use of restricted shuffling ensured that the histograms were all flat, as desirable, with no excesses of p-values at any range. Even for ise with skewed errors, a test that otherwise would be invalid, became valid on average and therefore useful in practice, although conservative. Power changes, though slight, are visible. Bland–Altman plots shown in the Supplementary Fig. 2 reveal that, even in the absence of dependence for data and for design, and without any simulated effect, the p-values for each test are not identical for unrestricted versus restricted shuffling, with the differences falling well outside the confidence interval for a much larger fraction of tests than the 5% that would be expected by chance.

7 It should be noted, however, that explicitly selecting permutations that maximise the amount of disarrangement applied to the data, i.e., performing only those with highest Hamming distance, causes the error rates not to be controlled; conversely, a selection of only those that causes less shuffling cause the test to become conservative (results not shown).

8 We have run a side analysis (not shown) in which we treated dz pairs as ordinary siblings, i.e., using the tree shown in Fig. 8 instead of the one in Fig. 7, and observed results that are very similar to those reported, that is, nothing significant.
