# Supplementary material for: Multi-level block permutation
Source: Neuroimage. 2015 Dec;123:253–68. doi: 10.1016/j.neuroimage.2015.05.092 (PMC4644991; doi:10.1016/j.neuroimage.2015.05.092)
Supplement: Supplementary file 1 — Supplementary material. [file mmc1.zip › SupplementaryMaterial.pdf]

Multi-level Block Permutation Supplementary Material

Anderson M. Winkler, Matthew A. Webster, Diego Vidaurre,
Thomas E. Nichols, Stephen M. Smith

Figure 1: *(on page 2)* Pictorial table showing the histograms of the distributions of p-values in different settings for the dataset A. In each histogram, the horizontal axis contains p-values in the range 0 to 1, split into 20 bins, while the vertical axis contains the relative frequencies. For the error rates, the vertical axis in the range 0 to 14%, in steps of 1%, and for power, in the range 0 to 100%, in steps of 10%. The error bars indicate one standard deviation on the height of each bar after 500 repetitions. To facilitate viewing, the bars for the error rates are shown in blue; for power, in green when the respective error rate was controlled at the nominal level of the test, or in red when the test became invalid. In general, when there is no actual dependence structure among either the data or model, the false positive rate was controlled; however, the tests became invalid when observations in both were not independent. This can be observed easily by noting that when $h^2_{\mathbf{m}}$ and $h^2_{\boldsymbol{\epsilon}}$ are both larger than zero, and in the absence of signal, there are excesses of very low and very high p-values. See the main text for details.

Figure 2: *(on page 3)* Pictorial table showing the Bland–Altman plots comparing the unrestricted with the restricted permutations for the dataset A. In each scatter plot, the horizontal axis contains average of the restricted and unrestricted p-values, in the range 0 to 1 and in steps of 0.1 in the marked grid, while the vertical axis contains the difference between the unrestricted and the restricted p-values, such that negative differences correspond to larger (less significant) restricted p-values. For the plots without effect, the vertical axis is in the range -0.3 to 0.3 and in steps of 0.1, and for those with effect, in the range -0.8 to 0.8 and in steps of 0.2. Because there would be too many p-values to be shown ($2.5 \cdot 10^5$, that is, 500 repetitions of 500 tests for each configuration), here only 1000 dots are shown on each of the 96 panels, two from each set at every repetition, selected randomly. To facilitate viewing, the dots for the error rates are shown in blue; for power, in green. The ellipsoids, shown in red, indicate the 95% confidence intervals.

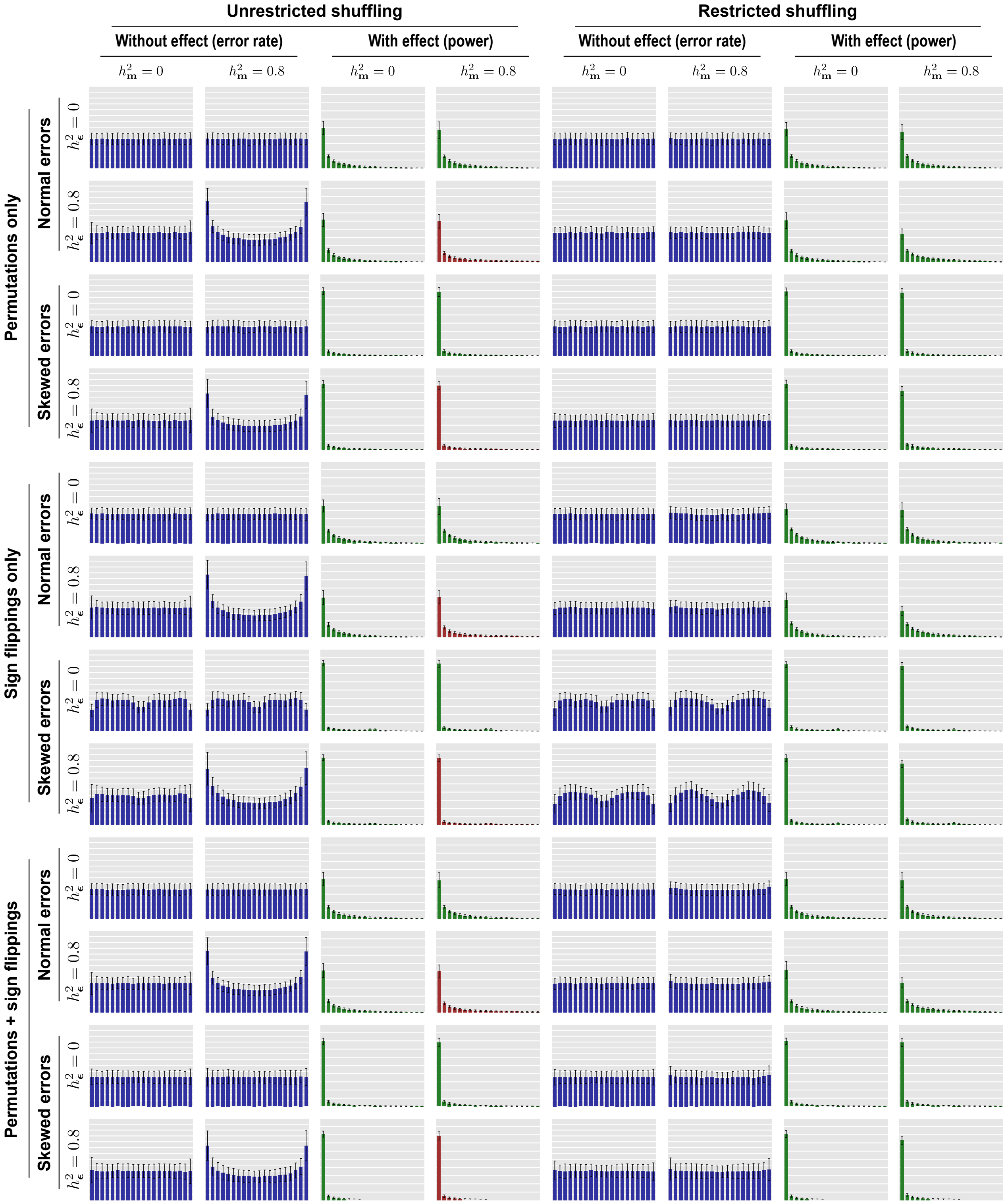

Unrestricted shuffling
Restricted shuffling
Without effect (error rate)
With effect (power)
Without effect (error rate)
With effect (power)
$h^2_{\mathrm{m}} = 0$
$h^2_{\mathrm{m}} = 0.8$
Permutations only
Sign flippings only
Permutations + sign flippings
Normal errors
Skewed errors
$h^2_{\epsilon} = 0$
$h^2_{\epsilon} = 0.8$

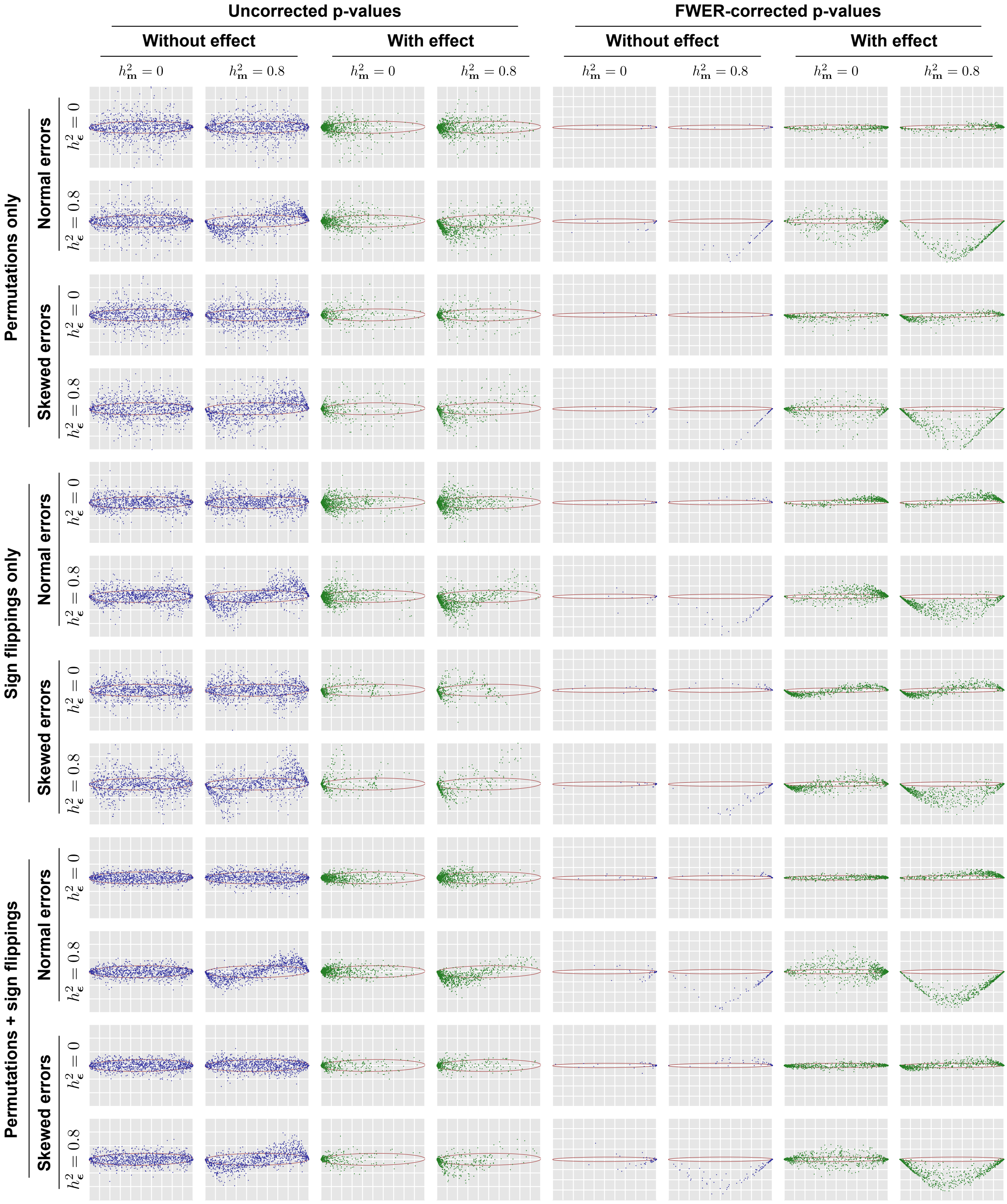

Uncorrected p-values
FWER-corrected p-values
Without effect
With effect
Without effect
With effect
$h^2_{\mathbf{m}} = 0$
$h^2_{\mathbf{m}} = 0.8$
Permutations only
Sign flippings only
Permutations + sign flippings
Normal errors
Skewed errors
$h^2_{\epsilon} = 0$
$h^2_{\epsilon} = 0.8$

Table 1: Proportion of error type I and power (%) for the simulated sets A and B, with Laplacian (kurtotic) errors, at the level $\alpha = 0.05$, using different degrees of dependence for the error terms (h^2_{ϵ}) and for the regressor of interest ($h^2_{\mathbf{m}}$), using permutations (EE), sign flippings (ISE), or permutations with sign flippings (EE+ISE). Confidence intervals (95%) are shown between parentheses. The values that appear ~~striked out~~ are not valid, as they refer to power observed when the corresponding error rates are not controlled (i.e., the lower bound of the confidence interval is above the nominal level α when there is no actual effect).

Set	h^2_{ϵ}	Unrestricted shuffling						Restricted shuffling					
		Without effect (error rate)			With effect (power)			Without effect (error rate)			With effect (power)		
		$h^2_{\mathbf{m}} = 0$	$h^2_{\mathbf{m}} = 0.4$	$h^2_{\mathbf{m}} = 0.8$	$h^2_{\mathbf{m}} = 0$	$h^2_{\mathbf{m}} = 0.4$	$h^2_{\mathbf{m}} = 0.8$	$h^2_{\mathbf{m}} = 0$	$h^2_{\mathbf{m}} = 0.4$	$h^2_{\mathbf{m}} = 0.8$	$h^2_{\mathbf{m}} = 0$	$h^2_{\mathbf{m}} = 0.4$	$h^2_{\mathbf{m}} = 0.8$
Permutations only:													
A	0.0	5.0 (3.4–7.3)	5.0 (3.4–7.3)	5.0 (3.4–7.3)	50.5 (46.1–54.8)	50.0 (45.6–54.3)	49.0 (44.6–53.3)	5.0 (3.4–7.3)	5.0 (3.4–7.2)	5.0 (3.4–7.3)	49.3 (44.9–53.7)	48.6 (44.2–53.0)	46.8 (42.4–51.1)
	0.4	4.9 (3.4–7.2)	6.3 (4.5–8.8)	7.7 (5.7–10.4)	51.7 (47.3–56.1)	50.7 (46.4–55.1)	~~49.8 (45.4–54.1)~~	5.0 (3.4–7.2)	4.9 (3.4–7.2)	5.0 (3.4–7.2)	50.7 (46.3–55.1)	45.3 (40.9–49.6)	39.9 (35.7–44.3)
	0.8	5.0 (3.4–7.2)	7.6 (5.6–10.3)	10.3 (8.0–13.3)	52.9 (48.5–57.3)	~~51.9 (47.5–56.2)~~	~~51.3 (46.9–55.7)~~	5.0 (3.4–7.3)	5.0 (3.4–7.3)	4.9 (3.4–7.2)	52.8 (48.5–57.2)	43.8 (39.5–48.2)	36.3 (32.2–40.6)
B	0.0	5.0 (3.4–7.2)	5.0 (3.4–7.3)	5.0 (3.4–7.2)	50.4 (46.0–54.7)	49.4 (45.1–53.8)	48.1 (43.7–52.4)	5.0 (3.4–7.3)	5.0 (3.4–7.3)	5.1 (3.5–7.4)	48.5 (44.2–52.9)	47.5 (43.2–51.9)	45.3 (41.0–49.7)
	0.4	4.9 (3.3–7.2)	6.2 (4.4–8.7)	7.5 (5.5–10.1)	51.9 (47.6–56.3)	50.8 (46.4–55.2)	~~50.0 (45.6–54.3)~~	5.0 (3.4–7.3)	5.0 (3.4–7.3)	5.0 (3.4–7.3)	50.4 (46.0–54.7)	45.4 (41.1–49.7)	40.0 (35.8–44.3)
	0.8	4.9 (3.3–7.1)	7.3 (5.4–10.0)	9.8 (7.5–12.8)	53.9 (49.5–58.2)	~~52.4 (48.0–56.7)~~	~~51.8 (47.4–56.1)~~	5.0 (3.4–7.3)	5.0 (3.4–7.3)	4.9 (3.3–7.1)	53.1 (48.8–57.5)	44.6 (40.3–48.9)	36.9 (32.8–41.2)
Sign flippings only:													
A	0.0	5.0 (3.4–7.2)	5.0 (3.4–7.3)	5.0 (3.4–7.2)	49.7 (45.4–54.1)	49.0 (44.6–53.4)	47.8 (43.5–52.2)	5.0 (3.4–7.3)	5.1 (3.5–7.4)	5.2 (3.6–7.5)	45.8 (41.5–50.2)	45.2 (40.8–49.5)	43.4 (39.1–47.8)
	0.4	4.9 (3.4–7.2)	6.4 (4.6–8.9)	7.8 (5.8–10.5)	50.6 (46.2–55.0)	50.5 (46.2–54.9)	~~49.2 (44.9–53.6)~~	4.9 (3.3–7.2)	5.0 (3.4–7.2)	5.1 (3.5–7.4)	46.7 (42.4–51.1)	43.1 (38.8–47.4)	38.0 (33.9–42.4)
	0.8	4.9 (3.3–7.2)	7.6 (5.6–10.3)	10.5 (8.1–13.5)	52.1 (47.7–56.4)	~~51.4 (47.1–55.8)~~	~~50.6 (46.2–55.0)~~	4.8 (3.3–7.1)	5.0 (3.4–7.3)	5.1 (3.5–7.4)	49.3 (44.9–53.7)	42.1 (37.8–46.5)	34.9 (30.9–39.2)
B	0.0	4.9 (3.3–7.1)	5.0 (3.4–7.2)	4.9 (3.3–7.2)	48.9 (44.5–53.2)	48.8 (44.4–53.1)	47.9 (43.6–52.3)	4.9 (3.3–7.2)	5.1 (3.5–7.4)	5.2 (3.6–7.5)	45.2 (40.9–49.6)	45.2 (40.9–49.6)	43.8 (39.5–48.1)
	0.4	5.0 (3.4–7.3)	6.2 (4.4–8.7)	7.6 (5.6–10.3)	49.4 (45.1–53.8)	49.9 (45.5–54.3)	~~49.8 (45.4–54.1)~~	4.9 (3.3–7.1)	5.0 (3.4–7.3)	5.2 (3.5–7.5)	45.8 (41.5–50.2)	43.4 (39.2–47.8)	38.7 (34.6–43.1)
	0.8	5.0 (3.4–7.3)	7.2 (5.2–9.8)	10.0 (7.7–13.0)	50.8 (46.4–55.2)	~~51.5 (47.1–55.8)~~	~~50.6 (46.3–55.0)~~	4.7 (3.2–7.0)	4.9 (3.3–7.1)	4.9 (3.4–7.2)	48.1 (43.7–52.5)	42.2 (38.0–46.6)	35.4 (31.4–39.7)
Permutations + sign flippings:													
A	0.0	4.9 (3.4–7.2)	5.0 (3.4–7.2)	5.1 (3.5–7.3)	50.6 (46.2–55.0)	49.3 (44.9–53.7)	48.5 (44.2–52.9)	4.9 (3.3–7.2)	5.1 (3.5–7.4)	5.3 (3.7–7.7)	50.3 (46.0–54.7)	49.4 (45.0–53.8)	48.6 (44.2–53.0)
	0.4	5.0 (3.4–7.3)	6.3 (4.5–8.8)	7.6 (5.6–10.3)	51.6 (47.2–55.9)	50.3 (46.0–54.7)	~~49.5 (45.2–53.9)~~	4.9 (3.4–7.2)	5.2 (3.6–7.5)	5.2 (3.6–7.6)	51.5 (47.1–55.8)	46.5 (42.1–50.8)	41.9 (37.6–46.2)
	0.8	5.0 (3.4–7.2)	7.9 (5.8–10.6)	10.2 (7.9–13.2)	52.8 (48.4–57.1)	~~52.9 (48.5–57.2)~~	~~51.9 (47.5–56.2)~~	5.0 (3.4–7.3)	5.2 (3.5–7.5)	5.3 (3.7–7.6)	53.2 (48.8–57.5)	45.7 (41.4–50.1)	38.6 (34.5–43.0)
B	0.0	5.1 (3.5–7.4)	5.0 (3.4–7.2)	4.9 (3.4–7.2)	51.1 (46.7–55.4)	50.3 (46.0–54.7)	48.4 (44.1–52.8)	5.0 (3.4–7.3)	5.2 (3.5–7.5)	5.3 (3.6–7.6)	50.8 (46.4–55.2)	50.4 (46.1–54.8)	48.4 (44.0–52.7)
	0.4	4.9 (3.3–7.2)	6.2 (4.4–8.6)	7.5 (5.5–10.2)	51.7 (47.3–56.0)	51.5 (47.1–55.8)	~~49.9 (45.5–54.3)~~	4.9 (3.3–7.1)	5.2 (3.6–7.5)	5.3 (3.7–7.6)	51.5 (47.1–55.9)	48.0 (43.7–52.4)	42.4 (38.2–46.8)
	0.8	4.9 (3.3–7.1)	7.4 (5.4–10.1)	10.1 (7.8–13.1)	53.5 (49.2–57.9)	~~52.1 (47.7–56.4)~~	~~51.4 (47.1–55.8)~~	5.0 (3.4–7.3)	5.2 (3.5–7.5)	5.4 (3.7–7.7)	53.8 (49.5–58.2)	45.9 (41.6–50.3)	38.6 (34.5–43.0)

Table 2: Proportion of error type I and power (%) for the simulated sets A and B, with Weibullian (skewed) errors, at the level $\alpha = 0.05$, using different degrees of dependence for the error terms (h^2_{ϵ}) and for the regressor of interest ($h^2_{\mathbf{m}}$), using permutations (EE), sign flippings (ISE), or permutations with sign flippings (EE+ISE). Confidence intervals (95%) are shown between parentheses. The values that appear ~~striked out~~ are not valid, as they refer to power observed when the corresponding error rates are not controlled (i.e., the lower bound of the confidence interval is above the nominal level α when there is no actual effect).

Set	h^2_{ϵ}	Unrestricted shuffling						Restricted shuffling					
		Without effect (error rate)			With effect (power)			Without effect (error rate)			With effect (power)		
		$h^2_{\mathbf{m}} = 0$	$h^2_{\mathbf{m}} = 0.4$	$h^2_{\mathbf{m}} = 0.8$	$h^2_{\mathbf{m}} = 0$	$h^2_{\mathbf{m}} = 0.4$	$h^2_{\mathbf{m}} = 0.8$	$h^2_{\mathbf{m}} = 0$	$h^2_{\mathbf{m}} = 0.4$	$h^2_{\mathbf{m}} = 0.8$	$h^2_{\mathbf{m}} = 0$	$h^2_{\mathbf{m}} = 0.4$	$h^2_{\mathbf{m}} = 0.8$
Permutations only:													
A	0.0	5.0 (3.4–7.3)	5.0 (3.4–7.3)	4.9 (3.4–7.2)	79.1 (75.3–82.4)	78.3 (74.5–81.7)	78.1 (74.2–81.5)	5.0 (3.4–7.3)	5.0 (3.4–7.3)	5.0 (3.4–7.2)	78.5 (74.7–81.9)	77.5 (73.7–81.0)	77.1 (73.3–80.6)
	0.4	5.1 (3.5–7.3)	6.3 (4.5–8.8)	7.7 (5.7–10.4)	79.4 (75.6–82.7)	78.6 (74.8–82.0)	~~77.7 (73.9–81.1)~~	4.9 (3.4–7.2)	5.0 (3.4–7.3)	5.0 (3.4–7.3)	78.9 (75.1–82.3)	76.3 (72.4–79.8)	73.3 (69.3–77.0)
	0.8	5.0 (3.4–7.3)	7.2 (5.2–9.8)	9.6 (7.3–12.5)	80.4 (76.7–83.7)	~~79.8 (76.0–83.1)~~	~~78.1 (74.3–81.5)~~	5.0 (3.4–7.3)	5.1 (3.5–7.4)	5.0 (3.4–7.3)	80.3 (76.6–83.5)	76.7 (72.8–80.2)	71.9 (67.8–75.6)
B	0.0	4.9 (3.3–7.2)	5.0 (3.4–7.2)	4.9 (3.3–7.1)	80.6 (76.9–83.8)	80.1 (76.3–83.3)	79.3 (75.5–82.6)	5.0 (3.4–7.3)	4.9 (3.4–7.2)	4.9 (3.4–7.2)	80.1 (76.4–83.4)	79.3 (75.5–82.6)	78.3 (74.5–81.7)
	0.4	5.0 (3.4–7.2)	6.2 (4.4–8.7)	7.5 (5.5–10.2)	81.2 (77.5–84.4)	80.7 (77.0–83.9)	~~79.5 (75.7–82.8)~~	4.9 (3.3–7.2)	5.0 (3.4–7.2)	5.0 (3.4–7.3)	80.6 (76.9–83.8)	78.7 (74.9–82.1)	75.5 (71.6–79.1)
	0.8	5.1 (3.5–7.4)	7.2 (5.2–9.8)	9.1 (6.9–12.0)	81.9 (78.3–85.1)	~~81.4 (77.7–84.5)~~	~~80.6 (76.9–83.8)~~	5.0 (3.4–7.3)	5.0 (3.4–7.3)	4.9 (3.4–7.2)	81.7 (78.1–84.8)	78.7 (74.9–82.1)	75.2 (71.2–78.8)
Sign flippings only:													
A	0.0	3.5 (2.2–5.6)	3.6 (2.3–5.6)	3.6 (2.3–5.6)	82.9 (79.3–85.9)	82.7 (79.2–85.8)	82.2 (78.6–85.3)	3.8 (2.5–5.9)	4.0 (2.6–6.0)	4.0 (2.6–6.1)	81.0 (77.4–84.2)	80.6 (76.9–83.8)	79.2 (75.4–82.5)
	0.4	4.2 (2.7–6.3)	5.9 (4.2–8.3)	7.8 (5.8–10.5)	82.3 (78.7–85.4)	82.1 (78.5–85.2)	~~81.3 (77.7–84.5)~~	3.7 (2.3–5.7)	3.7 (2.4–5.8)	3.8 (2.4–5.8)	81.0 (77.3–84.2)	78.9 (75.1–82.2)	75.7 (71.7–79.2)
	0.8	4.5 (3.0–6.7)	7.1 (5.1–9.6)	9.6 (7.3–12.5)	82.0 (78.4–85.2)	~~81.7 (78.0–84.8)~~	~~81.2 (77.6–84.4)~~	3.6 (2.3–5.6)	3.7 (2.4–5.7)	3.7 (2.3–5.7)	81.4 (77.7–84.5)	78.4 (74.6–81.8)	74.5 (70.5–78.1)
B	0.0	3.2 (2.0–5.1)	3.1 (1.9–5.1)	3.2 (1.9–5.1)	83.7 (80.2–86.7)	83.3 (79.8–86.3)	83.0 (79.5–86.1)	3.5 (2.2–5.5)	3.7 (2.3–5.7)	3.6 (2.3–5.6)	82.1 (78.5–85.3)	81.5 (77.8–84.6)	80.2 (76.5–83.4)
	0.4	3.7 (2.4–5.8)	5.3 (3.7–7.6)	6.6 (4.7–9.1)	83.2 (79.7–86.2)	82.7 (79.2–85.8)	82.2 (78.6–85.3)	3.4 (2.2–5.4)	3.5 (2.2–5.4)	3.5 (2.2–5.4)	82.0 (78.4–85.2)	80.0 (76.3–83.3)	77.0 (73.1–80.5)
	0.8	4.0 (2.6–6.1)	5.9 (4.2–8.4)	8.3 (6.2–11.0)	83.3 (79.8–86.3)	83.0 (79.5–86.0)	~~82.4 (78.9–85.5)~~	3.3 (2.1–5.3)	3.2 (2.0–5.1)	3.3 (2.1–5.3)	82.7 (79.2–85.8)	79.9 (76.2–83.2)	76.1 (72.1–79.6)
Permutations + sign flippings:													
A	0.0	5.0 (3.4–7.3)	5.0 (3.4–7.3)	5.0 (3.4–7.2)	79.3 (75.5–82.6)	78.5 (74.7–81.9)	78.0 (74.2–81.4)	5.0 (3.4–7.3)	5.2 (3.6–7.5)	5.3 (3.7–7.6)	79.3 (75.5–82.6)	78.7 (74.9–82.1)	78.1 (74.3–81.5)
	0.4	4.8 (3.3–7.1)	6.1 (4.4–8.6)	7.7 (5.7–10.3)	79.3 (75.6–82.6)	78.7 (74.9–82.0)	~~77.9 (74.1–81.4)~~	4.9 (3.3–7.1)	5.0 (3.4–7.3)	5.3 (3.7–7.7)	79.4 (75.7–82.7)	77.1 (73.2–80.5)	74.7 (70.7–78.3)
	0.8	5.1 (3.5–7.5)	7.3 (5.3–9.9)	9.3 (7.1–12.2)	80.7 (77.0–83.9)	~~79.9 (76.1–83.1)~~	~~78.6 (74.8–82.0)~~	5.1 (3.5–7.4)	5.3 (3.6–7.6)	5.3 (3.7–7.7)	80.9 (77.3–84.1)	77.2 (73.3–80.6)	73.2 (69.2–76.9)
B	0.0	4.9 (3.4–7.2)	5.0 (3.4–7.3)	5.0 (3.4–7.3)	80.5 (76.8–83.8)	80.3 (76.6–83.6)	79.5 (75.8–82.8)	4.9 (3.4–7.2)	5.2 (3.6–7.5)	5.4 (3.7–7.8)	80.5 (76.8–83.7)	80.3 (76.6–83.6)	79.4 (75.7–82.7)
	0.4	5.0 (3.4–7.3)	6.2 (4.4–8.6)	7.4 (5.4–10.0)	81.1 (77.4–84.3)	80.5 (76.8–83.7)	~~79.6 (75.8–82.9)~~	5.0 (3.4–7.2)	5.0 (3.4–7.3)	5.3 (3.7–7.7)	81.2 (77.5–84.4)	79.2 (75.4–82.5)	76.6 (72.6–80.1)
	0.8	5.1 (3.5–7.4)	7.1 (5.2–9.7)	9.0 (6.8–11.9)	82.0 (78.3–85.1)	~~81.5 (77.9–84.7)~~	~~80.4 (76.7–83.7)~~	4.9 (3.4–7.2)	5.1 (3.5–7.4)	5.3 (3.6–7.6)	82.1 (78.5–85.2)	79.3 (75.6–82.7)	75.7 (71.8–79.3)

Table 3: Peak significance levels for each of the correlations between height, weight and BMI, and cortical thickness and local cortical surface area. The p-values below $\alpha = 0.05$ are marked in **bold**; all values are corrected controlling the familywise-error rate (FWER) across the whole brain and across both positive and negative correlations. Permuting the data freely, therefore violating exchangeability, identified seemingly significant associations. However, using the proposed permutation strategy that respects the data structure, these findings disappear, suggesting that these significant results are, in fact, **false positives**. See also Figure 10 in the main text.

Trait	Cortical surface area				Cortical thickness			
	Unrestricted		Restricted		Unrestricted		Restricted	
	Left	Right	Left	Right	Left	Right	Left	Right
Positive correlation:								
Height	**0.0476**	0.2178	0.1530	0.4398	0.4168	0.0734	0.6438	0.1694
Weight	0.9524	0.7448	0.9724	0.8346	0.2348	0.1706	0.3118	0.2376
BMI	1.0000	0.9368	1.0000	0.9586	0.0950	**0.0340**	0.1376	0.0522
Negative correlation:								
Height	0.9674	0.9936	0.9944	0.9996	0.2206	**0.0306**	0.4204	0.0788
Weight	0.9960	0.9908	0.9992	0.9978	0.1246	**0.0462**	0.1860	0.0810
BMI	0.8002	0.9848	0.8644	0.9926	0.4492	0.2674	0.5620	0.3588
